# Reduction in Organic Aerosol from Coal Combustion is Partially Offset by Enhanced Secondary Formation during the Beijing Coal Burning Ban

**DOI:** 10.1021/acs.est.4c13051

**Published:** 2025-05-05

**Authors:** Haiyan Ni, Haobin Zhong, Ying Wang, Peng Yao, Jie Tian, Yongyong Ma, Ru-Jin Huang, Ulrike Dusek

**Affiliations:** † State Key Laboratory of Loess and Quaternary Geology, Key Laboratory of Aerosol Chemistry and Physics, CAS Center for Excellence in Quaternary Science and Global Change, Institute of Earth Environment, Chinese Academy of Sciences, Xi’an 710061, China; ‡ School of Advanced Materials Engineering, Jiaxing Nanhu University, Jiaxing 314001, China; § Centre for Isotope Research (CIO), Energy and Sustainability Research Institute Groningen (ESRIG), University of Groningen, Groningen 9747AG, the Netherlands; ∥ School of Environmental and Municipal Engineering, Xi’an University of Architecture and Technology, Xi’an 710055, China; ⊥ Meteorological Institute of Shaanxi Province, Xi’an 710015, China

**Keywords:** coal ban, reduced coal combustion emission, organic aerosol, radiocarbon analysis, ACSM-PMF analysis, weather normalization technique

## Abstract

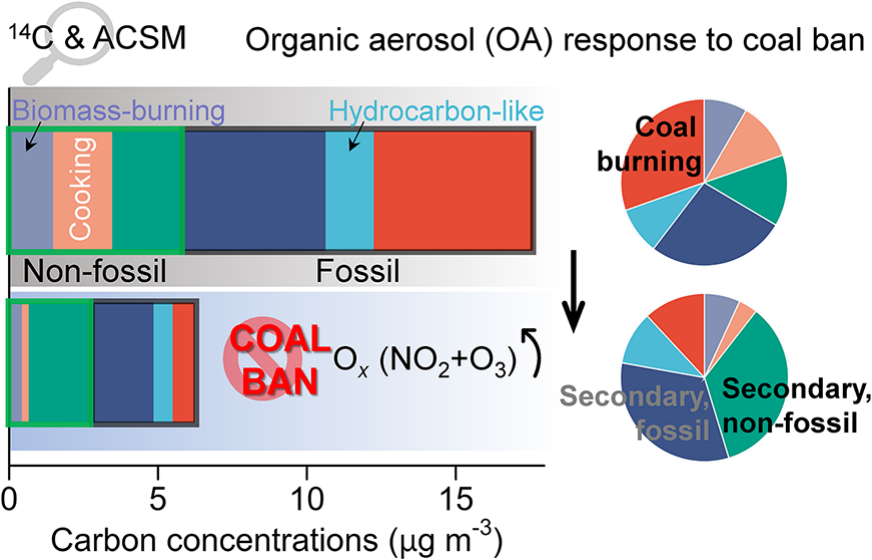

A coal ban policy in northern China during winter 2017 enforced a switch from coal to gas or electricity for residential heating, providing a unique opportunity to study the effect of reduced coal combustion emissions on organic aerosol (OA). This study explores OA composition, sources, and atmospheric transformations in Beijing before and during the coal ban using online aerosol chemical speciation monitor (ACSM) and offline ^14^C measurements. Four primary factors (hydrocarbon-like, cooking, biomass burning, coal combustion OA) and one secondary factor (oxygenated OA, OOA) were resolved from ACSM. In response to the coal ban, OA concentrations generally decreased, but coal combustion OA decreased most strongly, consistent with the decreased fossil carbon contributions to OA (67 ± 3% before vs 55 ± 4% during the ban). Concurrently, the OOA fraction increased from 45 to 72%, due to a larger decrease in concentrations of primary OA (POA; 59–88%) compared to OOA (34%), highlighting the enhanced secondary aerosol formation during the coal ban period. This aligns with the ^14^C evidence of higher water-soluble carbon in fossil OA (which has mostly secondary sources). During the coal ban period, O_*x*_ concentrations doubled and were positively correlated with the OOA fraction, highlighting strong photochemical OA production. The results show that the reduction of POA from stringent clean air actions is partially offset by enhanced secondary OA formation.

## Abstract 2

China’s coal ban effectively reduced primary OA emissions from coal combustion but triggered a rise in secondary OA formation, as evidenced by ACSM and ^14^C analysis

## 1 Introduction

Reducing coal combustion in China, where more than one-third of homes rely on coal stoves for heating and cooking,^1^ could eliminate a major source of primary emissions and secondary formation of fine aerosol particles (also known as particulate matter or PM), thus benefiting air quality and public health.^2−4^ Aimed at alleviating PM pollution and associated health impacts, a coal ban policy was introduced in 2017 in Beijing and its neighboring provinces. This policy promoted the substitution of household coal with natural gas and electricity, updated outdated industrial capacity standards, and eliminated small industrial coal-fired boilers.^5^ These stringent coal ban measures markedly reduced emissions from coal combustion and were found to contribute significantly to total PM reduction and related PM mortality in winter 2017/18 compared to the same periods before the implementation of coal ban,^6−8^ demonstrating the effectiveness of the coal ban policy. However, only a few studies investigated the influence on organic aerosol (OA),^9,10^ a dominant PM component with complex sources and atmospheric processes. OA includes primary OA (POA) that is directly emitted from sources (e.g., coal combustion, biomass burning, traffic) and secondary OA (SOA) formed in the atmosphere from precursor gases through complex atmospheric processes involving in photochemical reactions and aqueous chemistry.^11−13^ The mass fraction of SOA in total OA is large in Beijing and other Chinese megacities, constituting up to 80% of the total OA.^14−16^ This stresses the importance to study the sources and atmospheric processes of OA, especially SOA.^15,17^

High-resolution time-of-flight aerosol mass spectrometers (AMS) and aerosol chemical speciation monitors (ACSM) have improved our understanding of chemical composition and sources of OA.^18,19^ Combining AMS or ACSM mass spectra with factor analysis such as positive matrix factorization analysis (PMF) has been widely used to study the sources and formation of OA.^16,20^ Using ACSM-PMF, different types of organic factors, including various POA and SOA factors corresponding to different primary sources and secondary processes, have been identified. For example, Sun et al.^21^ found that primary coal combustion OA (CCOA) and oxygenated OA (OOA, a mix of SOA and other oxidized OA) dominated total OA mass, and photochemical processing played an important role in OOA production in Beijing during wintertime in 2011, before the implementation of the coal ban policy. However, fossil and nonfossil sources of OOA are still largely unquantified by AMS-PMF or ACSM-PMF methods.

Radiocarbon (^14^C) analysis is a powerful tool for separating fossil from nonfossil source contributions to OA because fossil OA sources (primarily coal and liquid fossil fuel) are completely depleted in ^14^C (half-life of 5730 years), whereas nonfossil OA sources (e.g., biomass burning, cooking, or biogenic emissions) contain a contemporary ^14^C signature.^22−27^ Moreover, a detailed ^14^C-based source apportionment can be obtained when ^14^C measurements are performed on different carbon fractions of OA, including total carbon in OA (OC) and water-soluble carbon in OA (WSOC).^28−30^ Combining ^14^C analysis with the AMS-PMF or ACSM-PMF results can constrain the fossil versus nonfossil carbon contributions to OOA.^31−34^ For example, based on this method, roughly equal contributions of fossil and nonfossil emissions to OOA were found in winter in Krakow, Poland.^35^

Changes of OA characteristics in Beijing during the implementation of the strict coal ban policy are still understudied, especially in terms of quantitative distinction between OOA formed from fossil and nonfossil precursors, which allows much more accurate tracing of reductions attributable to the coal ban. The coal ban policy and the resulting remarked reduction in emissions in winter 2017 serve as a natural experiment to evaluate the responses of OA sources and to assess the interplay between emission and atmospheric chemistry of OA. The main purpose of this study is to assess how the coal ban policy impacts POA and OOA in Beijing. A combination of ACSM factor analysis and ^14^C measurements allows us to trace the nonlinear effects of reducing a single emission source (coal burning) on OA concentrations, as well as changes in OA formation and oxidative processing. The results will help in developing effective air pollution control strategies in China.

## 2 Methods

### 2.1 Aerosol Sampling

Aerosol filter samples were collected on the roof of a five-story building (∼20 m) at the National Centre for Nanoscience (39.99 °N, 116.32 °E), an urban background site in Beijing. The 24 h (10:00 to 10:00 the following day, China standard time) samples were collected on precombusted quartz fiber filters (QM-A, Whatman Inc.) in two winters of 2016/17 and 2017/18. From 2 December 2016 to 8 January 2017, PM_2.5_ samples were collected using a high-volume aerosol sampler with a flow rate of 1.0 m^3^ min^–1^ (TE-6070 MFC, Tisch Inc.). Between 29 December 2017 to 12 March 2018, size-resolved aerosol samples were collected using a 5-stage high-flow impactor (flow rate of 100 L min^–1^; Model 130, Copley Scientific) with cutoff sizes of 2.5, 1.4, 0.77, 0.44, and 0.25 μm. After sampling, each filter sample was packed separately and stored in a −18 °C freezer. Detailed sampling information can be found in our previous studies.^36,37^ A subset of the collected samples was selected for the ^14^C measurements.

### 2.2 OC/EC Mass Quantification

For PM_2.5_ samples, OC and elemental carbon (EC) mass concentrations were determined using a carbon analyzer (DRI Model 2001, Atmoslytic Inc.) following the IMPROVE_A thermal–optical protocol.^38^ For size-resolved aerosol samples with cutoff sizes of 0.25 μm and higher, only total carbon (TC = OC + EC) mass was measured because the nonuniform impactor deposits on the filter hinder the determination of the OC/EC split point. OC and EC mass was therefore estimated from the extracted OC and EC mass for ^14^C analysis (Section 2.3) and the corresponding recoveries for particles with *D*_p_ < 0.25 μm (that deposit uniformly on the backup filter) from the same set of size-resolved samples following the method detailed in Ni et al.,^36^ resulting in high uncertainties of ∼20% for both OC and EC.

### 2.3 Radiocarbon Analysis

For the ^14^C analysis, we selected a subset of offline filter samples with varying aerosol loadings to represent different pollution conditions. For winter 2017/18, the selected four samples cover 6 days including three 24 h samples and one composite sample combining three 24 h samples with similar PM_2.5_ concentrations (difference within 15%) and back trajectories. For winter 2016/17, six samples were selected: five were 24 h samples, and one was a composite sample combining two daily samples. The observed ^14^C values do not vary significantly across the pollution conditions, with PM_2.5_ concentrations ranging from 43 to 175 μg m^–3^ in winter 2017/18 and from 12 to 357 μg m^–3^ in winter 2016/17 (Table S1). This further increases the credibility that the selected samples represent ^14^C signatures for the winter season.

EC, OC, and water-insoluble OC (WIOC) in aerosol samples were converted to CO_2_ using an aerosol combustion system (ACS)^39^ following the isolation protocol summarized in Supporting Text S1.^40,41^ The ACS system employed a reduction oven filled with copper grains and silver, a dry ice–ethanol bath and a flask filled with phosphorus pentoxide to remove possible interfering gases (e.g., NO_*x*_, halogens, and water vapors) from CO_2_. For ^14^C measurements, the extracted CO_2_ was converted to graphite and analyzed with accelerator mass spectrometry (^14^C-AMS) obtained from High Voltage Engineering^42,43^ or directly introduced into a gas ion source of the Mini Carbon Dating System (MICADAS; Ionplus AG) ^14^C-AMS at the University of Groningen.^44^ The transition from the old ^14^C-AMS to the new MICADAS has little impact on the ^14^C results.

^14^C data are reported using fraction modern (F^14^C) following the nomenclature of Reimer et al.^45^ F^14^C relates the sample ^14^C/^12^C ratio to the ^14^C/^12^C ratio of the standard OXII. Both of the ratios are normalized to δ^13^C = −25‰ to eliminate the effect of isotope fractionation
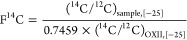
1

The F^14^C values were corrected for contamination during sample preparation and ^14^C-AMS measurements. The contamination was quantified from the F^14^C measurements of standards, including OXII with nominal F^14^C of 1.3407 and Rommenhöller with nominal F^14^C of zero.^46,47^ Secondary standards IAEA-C7 and IAEA-C8^48^ were measured in the same measurement series for quality control purpose. The measured F^14^C values (0.495 ± 0.008 for IAEA-C7 and 0.154 ± 0.007 for IAEA-C8) were found to agree with their respective consensus values within the uncertainties, showing the reliability of ^14^C measurements.

For size-resolved aerosol samples in winter 2017/18, F^14^C of EC, OC, and WIOC was directly measured. F^14^C of WSOC was calculated from the mass and isotopic differences between OC and WIOC (eq S3 in Supporting Table S2). Mass concentrations of WIOC were estimated from the extracted WIOC mass for ^14^C analysis, assuming that the recovery of WIOC varies between the OC recovery and 100% (i.e., WIOC is completely extracted), following the method detailed by Dusek et al.^40^ F^14^C values for total PM_2.5_ were estimated as a weighted average of the F^14^C values measured for different particle size ranges (<0.25, 0.25–0.44, 0.44–0.77, 0.77–1.4, and 1.4–2.5 μm). For PM_2.5_ samples in winter 2016/17, F^14^C of EC and OC was determined and cited from our previous study for comparison (Figure S1).^37^

### 2.4 ^14^C-Based Source Apportionment of EC and OC

F^14^C of EC, OC, WIOC, and WSOC allowed us to unambiguously apportion their fossil versus nonfossil sources. Nonfossil carbon fractions in EC, OC, WIOC, and WSOC (*f*_bb_(EC), *f*_nf_(OC), *f*_nf_(WIOC), *f*_nf_(WSOC)) were calculated from their F^14^C values by dividing their respective F^14^C of nonfossil sources (F^14^C_nf_) to correct the influence of the excess ^14^C from nuclear bomb tests in the 1960s. F^14^C_nf_ was estimated to be 1.10 ± 0.05 for EC and 1.09 ± 0.05 for OC fractions, as detailed by Ni et al.^26^ Given the nonfossil fraction, the fossil carbon fractions (*f*_fossil_(EC), *f*_fossil_(OC), *f*_fossil_(WIOC), *f*_fossil_(WSOC)) were calculated by *f*_fossil_ = 1 – *f*_nf_. Carbon mass from nonfossil sources (EC_bb_, OC_nf_, WIOC_nf_, WSOC_nf_) and from fossil sources (EC_fossil_, OC_fossil_, WIOC_fossil_, WSOC_fossil_) were determined using eqs (S6–S13) in Table S2. The term “EC_bb_” is used for nonfossil EC because biomass burning is the only nonfossil source for EC, but OC also has other nonfossil sources, e.g., cooking and biogenic emissions. The final uncertainties of the ^14^C source apportionment results were propagated from the uncertainties in carbon mass, F^14^C, and F^14^C_nf_ of different carbon fractions by conducting a Monte Carlo simulation (*n* = 10,000) following eqs (S3–S13), as detailed in our previous studies.^27^

### 2.5 ACSM Operation and OA Source Apportionment

At the same location of aerosol filter sampling, the chemical composition of nonrefractory PM_2.5_ including organics was measured online using a time-of-flight ACSM (ToF-ACSM; Aerodyne Research Inc.) with a time resolution of 2 min in winter 2017/18 (3 February 2018–15 March 2018). The detailed setup and operations of the ToF-ACSM is presented by Zhong et al.^49,50^ The OA source apportionment was performed by PMF and multilinear engine (ME-2) analysis of OA mass spectra obtained from ToF-ACSM observations (hereafter referred to as the ACSM-PMF method for OA source apportionment).^51^ After careful analysis of mass spectra, time series of each factor, and comparison with factors from previous studies, five factors were resolved: hydrocarbon-like OA (HOA), cooking OA (COA), biomass-burning OA (BBOA), CCOA, and OOA. A more detailed description of PMF and ME-2 analysis and OA source apportionment methods is provided in Supporting Text S2 and Figures S2–S7.

Source apportionment results of OA in nonrefractory PM_1_ at the same location in winter 2015/16 (3 February 2016–15 March 2016) from our previous published work were used for comparison in this study.^52^ The technical comparability of ACSM measurements between the two winters is detailed in Supporting Text S3. It is noteworthy that the ACSM-PMF was applied to PM_2.5_ samples in winter 2017/18 but to PM_1_ samples in winter 2015/16. This may consequently cause some uncertainties when comparing the ACSM-PMF results between the two winters. However, despite lower absolute OA concentrations in PM_1_ than in PM_2.5_, previous studies found no significant difference in OA composition (i.e., the contribution of different OA factors to the total OA mass) between PM_1_ and PM_2.5_, except during fog period with RH = 100% (which was not observed in this study).^53,54^ Therefore, our comparison of the ACSM-PMF results, particularly with respect to OA composition, between the two winters is still largely valid.

### 2.6 Coupling of ACSM-PMF and ^14^C Analysis of OC

This research defines two study periods: the “coal ban (CB) period” in winter 2017/18, when coal bans were enacted in Beijing, and the “pre-CB period”, which refers to the wintertime periods before policy implementation. To integrate the results of the ACSM-PMF with the ^14^C data of OC for each period, we first calculated averages over the entire data sets available for the pre-CB and CB periods. Specifically, the average for the ACSM-PMF results over the continuous sampling periods (CB: 3 February 2018–15 March 2018; pre-CB: 3 February 2016–15 March 2016) was calculated. For the ^14^C of OC, winter averages of its ^14^C results were used (see discrete sampling dates in Table S1). These period-specific averages were used for the combined source apportionment with the justification as follows. Despite the limited ^14^C data points, they are representative for CB and pre-CB winters, covering different time periods and pollution levels. For the CB period, the general sampling time periods of ^14^C data (29 December 2017–12 March 2018; see selected samples for ^14^C analysis in Table S1) overlap with ACSM (3 February 2018–15 March 2018) in winter 2017/18. The limited CB ^14^C data of OC do not vary significantly (*f*_fossil_(OC) = 0.50–0.57) with PM_2.5_ concentrations (43–175 μg m^–3^; Table S1), supporting the reliability of the selected samples in representing the ^14^C signatures for the CB period. For the pre-CB period, no ^14^C data of OC are available for the ACSM period (3 February 2016–15 March 2016), so we combine our previous study (December 2016 and January 2017)^37^ with data from the literature (January 2013 and January 2014; Table S1) for a pre-CB ^14^C estimate. These ^14^C values of OC show relatively stable values over the pre-CB winters (*f*_fossil_(OC) = 0.65 ± 0.05). When combining the ^14^C data of OC with the ACSM data, we notice that most of the ^14^C data were from December to January, while the ACSM data were from February to March; that is, the ^14^C and ACSM data were from different winter months for the pre-CB period. While not concurrent for the pre-CB period, combining the different winter months of ACSM and ^14^C is reasonable, given that available Beijing ^14^C data of OC shows limited ^14^C variability in winter months between December and January compared to February and March (Supporting Text S4). While this approach is less accurate than only using concurrent ACSM and ^14^C measurements, we argue that the pre-CB and CB averages are still representative for these periods.

**Figure 1:**
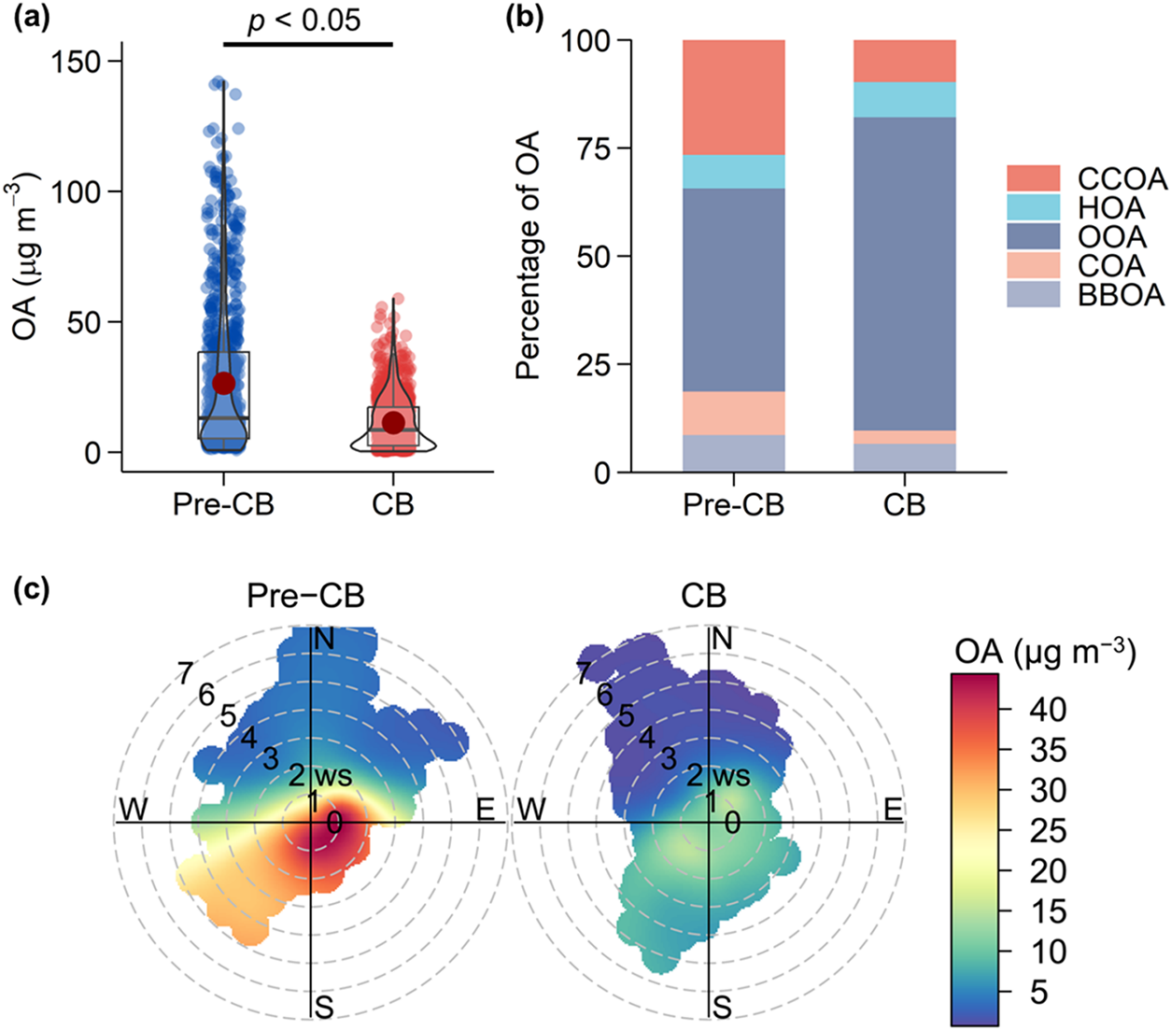
(a) A violin plot showing the distribution of organic aerosol (OA) concentrations from 3 February 2018 to 15 March 2018 during the implementation of coal ban (CB) measures in winter 2017/18, compared to the winter period from 3 February 2016 to 15 March 2016 before the CB (pre-CB); (b) contribution of different OA factors to the total OA mass during the pre-CB and CB periods; (c) bivariate polar plot that illustrates the variation of OA concentrations as a function of wind speed (m s^–1^) and wind direction during the pre-CB and CB periods. *P* values determined by *t* tests at 95% confidence level are shown on top of panel (a). In panel (b), OA factors include coal combustion OA (CCOA), hydrocarbon-like OA (HOA), oxygenated OA (OOA), cooking OA (COA), and biomass-burning OA (BBOA).

Further, the OA concentrations from ACSM-PMF (Figures 1 and S2) need to be converted to OC concentrations based on source-specific OA/OC ratios in Beijing from the literature, where available, to align with the OC-based ^14^C results. These ratios were 1.35 for CCOA/CCOC (coal combustion OC), 1.31 for HOA/HOC (hydrocarbon-like OC), 1.38 for COA/COC (cooking OC), 1.58 for BBOA/BBOC (biomass-burning OC), and 1.78 for OOA/OOC (oxygenated OC).^55−57^

Then, the OC factors obtained from ACSM-PMF were divided into fossil and nonfossil fractions. HOC and CCOC were exclusively assigned to fossil sources, with the assumption of a negligible fraction of biofuel added to liquid fossil fuel (e.g., gasoline and diesel) for vehicles. BBOC and COC are considered to originate from nonfossil sources. To explore the fossil and nonfossil contribution to OOC, the ^14^C-determined fossil and nonfossil OC concentrations (OC_fossil_ and OC_nf_, respectively; see Section 2.4) were employed to apportion OOC into two distinct fractions, OOC of fossil and nonfossil origins (OOC_fossil_ and OOC_nf_, respectively), using the following equations
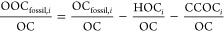
2
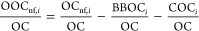
3

To estimate uncertainties of the fractions of the OOC_fossil_ and the OOC_nf_ in total OC (OOC_fossil_/OC and the OOC_nf_/OC, respectively), a Monte Carlo simulation of 10,000 runs was conducted to propagate uncertainties. For each run *i*, input parameters are randomly chosen from a normal distribution. Uncertainties of HOC/OC, CCOC/OC, BBOC/OC, and COC/OC arise from the source-specific ratio of OA to OC and ACSM-PMF. Currently, no universally established and widely accepted methodology exists for evaluating the uncertainties of ACSM-PMF results. Here, we accounted for the inherent ambiguities arising from the selection of the number of factors (10%).^58^ Uncertainties of OC_fossil_/OC and OC_nf_/OC, i.e., ^14^C-determined *f*_fossil_(OC) and *f*_nf_(OC), are propagated from F^14^C and F^14^C_nf_ for OC in Section 2.4. The uncertainties (RSD) of OOC_fossil_/OC and OOC_nf_/OC were estimated as on average 14 and 18%, respectively.

### 2.7 Random Forest (RF) Model and Weather Normalization

A machine-learning-based RF algorithm model, combined with the OA source apportionment results, was employed to decouple the effects of weather conditions (meteorological data and air mass clusters) on variations in concentrations of OA factors. To build an RF model for each OA factor, a data set was prepared as input predictor features, including time variables (Unix time, Julian day, month, week of the year, day of the week, hour of the day), observed meteorological data (relative humidity, wind speed, wind direction, temperature), other meteorological data (boundary layer height, precipitation, surface pressure, total cloud cover) from ERA5 reanalysis data set, and air mass clusters categorized by the HYSPLIT backward trajectories on the basis of Euclidean distance. To calculate deweathered concentrations, the weather normalization resampled the weather variables from the whole study period and was randomly allocated to a dependent variable observation. The detailed methodology, including RF model parameters, model performance, and the weather normalization procedure, is provided in Text S5 and Table S3.

## 3 Results and Discussion

### 3.1 Changes in OA Characteristics

The coal ban (CB) measures were implemented in Beijing in early 2017. This splits our campaigns into two periods, including the CB period in winter 2017/18 and the wintertime periods before the CB (hereafter referred to as the pre-CB period). In response to the CB measures, the mass concentrations of OA dropped dramatically from 26.4 μg m^–3^ during the pre-CB period to 11.3 μg m^–3^ during the CB period (Figure 1a), a decrease of 57%. All OA factors decreased from pre-CB to CB period, with primary OA factors (CCOA, HOA, COA, BBOA) showing more substantial decreases (59–88%) than the secondary factor (OOA), which decreased by 34% (Figure S8). Among the OA factors, CCOA, the primary OA from coal combustion, showed the largest decrease by 6.1 μg m^–3^. The strong decrease in CCOA, along with the decrease in SO_2_ concentrations (Figure S9 and Text S6), which is primary emitted from coal combustion, shows that the CB measures were effective and played a positive role in control of aerosol pollution in Beijing. During the CB period, CCOA on average accounted for 10% of the OA mass, which was much lower than that during the pre-CB period (28%; Figure 1b). In contrast, OOA was the most abundant OA factor during both pre-CB and CB periods, with its mass fraction in total OA increasing from 45% (pre-CB) to 72% (CB). Other OA factors, including HOA, COA, and BBOA, showed small to moderate changes in terms of their mass factions in total OA. These variations suggest reduced primary emissions (particularly, primary emissions from coal combustion) and enhanced secondary aerosol formation during the CB period. Similar findings were also observed during the COVID-19 lockdown period and the staggered-peak production periods,^52,59^ when various stringent emission controls were being implemented to improve the air quality. Pre-CB and CB exhibited similar characteristics in their bivariate polar plots, namely, that highest OA concentrations mainly occurred at a low wind speed of <3 m s^–1^, while at higher wind speeds of 3–6 m s^–1^, elevated concentrations occurred with southerly wind. This demonstrated that elevated OA in both pre-CB and CB was mainly dominated by local emissions, and in addition due to transport from the south of Beijing, which was also found in previous observation studies in Beijing.^60,61^

### 3.2 Formation of Secondary OA

**Figure 2:**
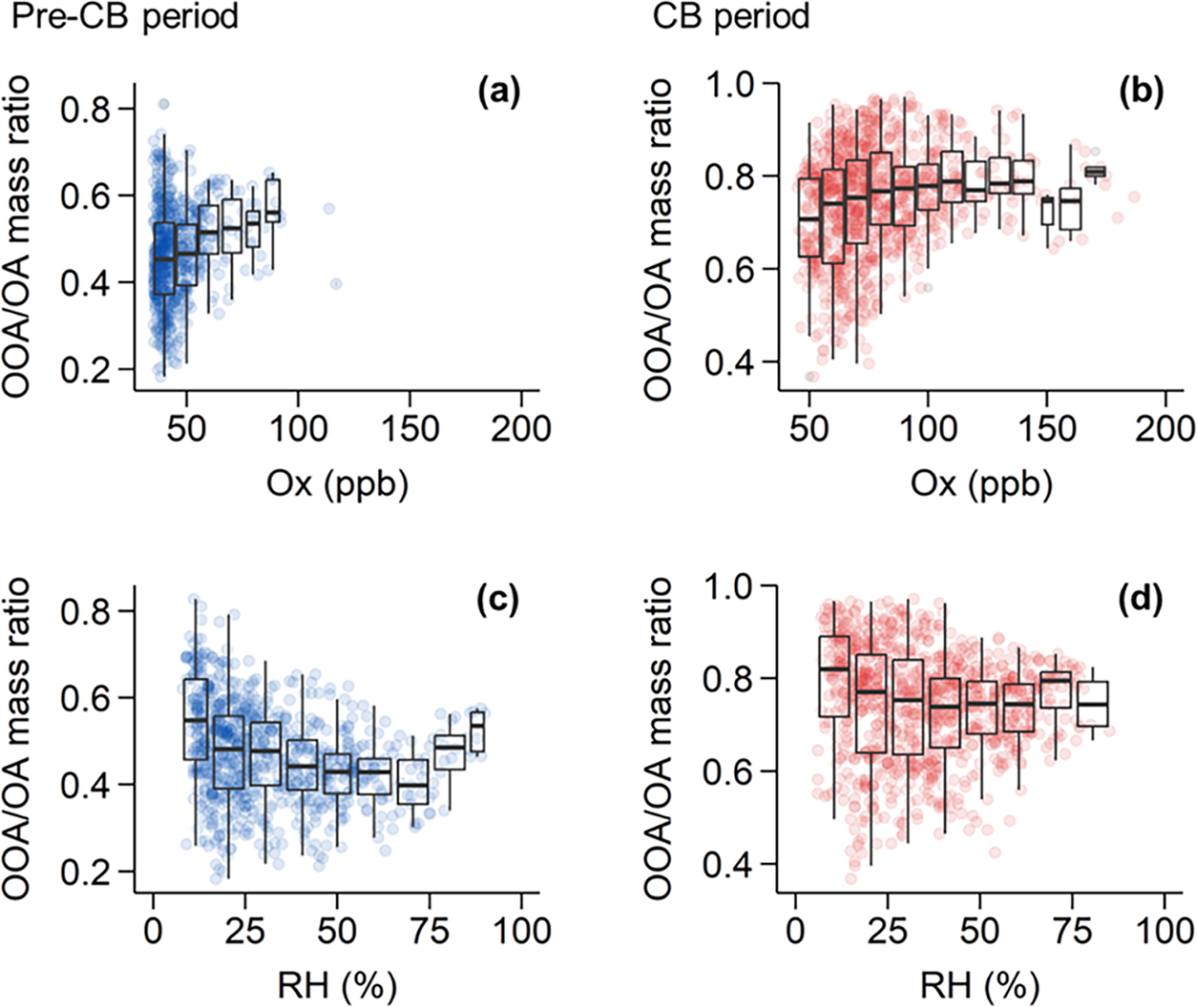
Mass fraction of OOA in total OA (i.e., OOA/OA) as functions of O_*x*_ (the sum of O_3_ and NO_2_) concentrations and RH during the pre-CB (a, c) and CB periods (b, d). Data were binned according to the RH (ΔRH = 10%) and the O_*x*_ concentrations (ΔO_*x*_ = 10 ppb).

To elucidate the pathways of secondary OA formation for the pre-CB and CB periods, the relationships between the mass fraction of OOA in total OA and O_*x*_ concentration (O_*x*_ = O_3_ + NO_2_) as well as RH are shown in Figure 2, with widely used O_*x*_ and RH as indicators of the extent of photochemical processing and aqueous-phase reaction, respectively.^59,62^ Using the OOA fraction better reflects the extent of secondary OA formation independent of the mass concentrations that can be influenced by changes in primary emissions or meteorological conditions. During the pre-CB period, the mass fraction of OOA in total OA showed an increase from 45 to 56% with the increase in O_*x*_ concentrations from 40 to 88 ppb (Figure 2a), suggesting that photochemical oxidation played an important role in OOA formation in winter of Beijing. As for the response to changes in RH, the mass fraction of OOA decreased with increasing RH at RH < 70% first and then increased with RH under high RH of >70% (Figure 2c). At low RH of <70%, both OOA and POA concentrations increased with RH (Figures S10 and S12), and the decreasing mass fraction of OOA is due to smaller increase of OOA concentrations relative to POA as RH increased. Possible explanations include (i) POA concentrations, being directly emitted and distributed throughout the boundary layer, are usually higher at lower boundary heights. In turn, boundary layer heights are often anticorrelated with RH (Figure S13), leading to higher POA concentrations at higher RH; and (ii) OOA formation is limited by secondary processes, such as aqueous-phase reactions, which are less active at lower RH. In contrast, at RH > 70%, the mass fraction of OOA increased with RH. Taken together, this suggests that aqueous-phase processing became more significant under high RH conditions, facilitating the formation of OOA, along with the photochemical oxidation during the pre-CB period.

The CB period had much higher O_*x*_ concentrations but similar RH values in comparison to the pre-CB period. During the CB period, the mass fraction of OOA increased from 71 to 78% as O_*x*_ concentrations increased from 50 to 100 ppb, with a similar increase rate as that during the pre-CB period, indicating equally efficient formation of OOA under the conditions of O_*x*_ < 100 ppb for the two periods. However, it did not show a further increase as the level of O_*x*_ surpasses 100 ppb during the CB period. This suggests that the production of OOA becomes less dependent on O_*x*_ levels when O_*x*_ > 100 ppb, likely due to the availability of OOA precursors or other limiting factors in the atmospheric processes. OOA mass concentrations during the CB period, notably lower than those during the pre-CB period, increased with increasing O_*x*_ concentrations in the whole range (Figure S10). The mass fraction of OOA showed no clear increasing or decreasing trends with RH, but OOA concentrations showed an increasing trend with the increase of RH during the CB periods (Figure S10). POA factors showed a similar increasing trend with RH (Figures S11 and S12), and this suggests the increase of both OOA and POA with RH is likely due to meteorology, e.g., lower boundary layer. Based on the above-discussed relationships between the OOA fraction with O_*x*_ and RH, photochemical reactions associated with high O_*x*_ concentrations may play a dominant role in the OOA formation during the CB period, surpassing the role of aqueous-phase chemistry.

Overall, our observations highlight the complex nature of atmospheric processes that photochemical oxidation and aqueous-phase reaction exerted a combined influence on the OOA formation during winter in Beijing and that the larger mass fraction of OOA in OA during the CB period was mainly driven by the enhanced photochemical oxidation associated with higher O_*x*_ concentrations relative to the pre-CB period (79 ± 25 ppb vs 40 ± 13 ppb). The increased level of O_*x*_ during the CB period cannot be attributed to the changes in solar radiation, as no increase in surface net solar radiation was found from the pre-CB to the CB periods (Figure S14). The enhanced atmospheric oxidation capacity during the CB period was also evidenced from the higher fraction of O_3_ in O_*x*_ during the CB period (on average of 0.54 vs 0.45 during the pre-CB period). This is consistent with previous studies in the same area, which have found that the declined fine particle levels—resulting from control measures and reduction in anthropogenic emissions (e.g., COVID-19 lockdown, “Air Pollution Prevention and Control Action Plan (2013–2017)”)—often induce O_*x*_ enhancement.^62,63^ Since the O_3_ formation mechanisms may differ depending on the specific emission reduction scenarios, the O_*x*_ increase during the CB period needs further investigation. However, for the purpose of this study, we can conclude that the observed O_*x*_ enhancements contribute to enhanced atmospheric oxidizing capacity and secondary aerosol formation. Our results show that reducing only one source (coal combustion) alone substantially decreased overall OA concentrations, and limiting emissions from this single source can influence the atmospheric oxidant levels and as a consequence increase SOA formation.

### 3.3 Changes in ^14^C of EC, OC, WIOC, and WSOC

**Figure 3:**
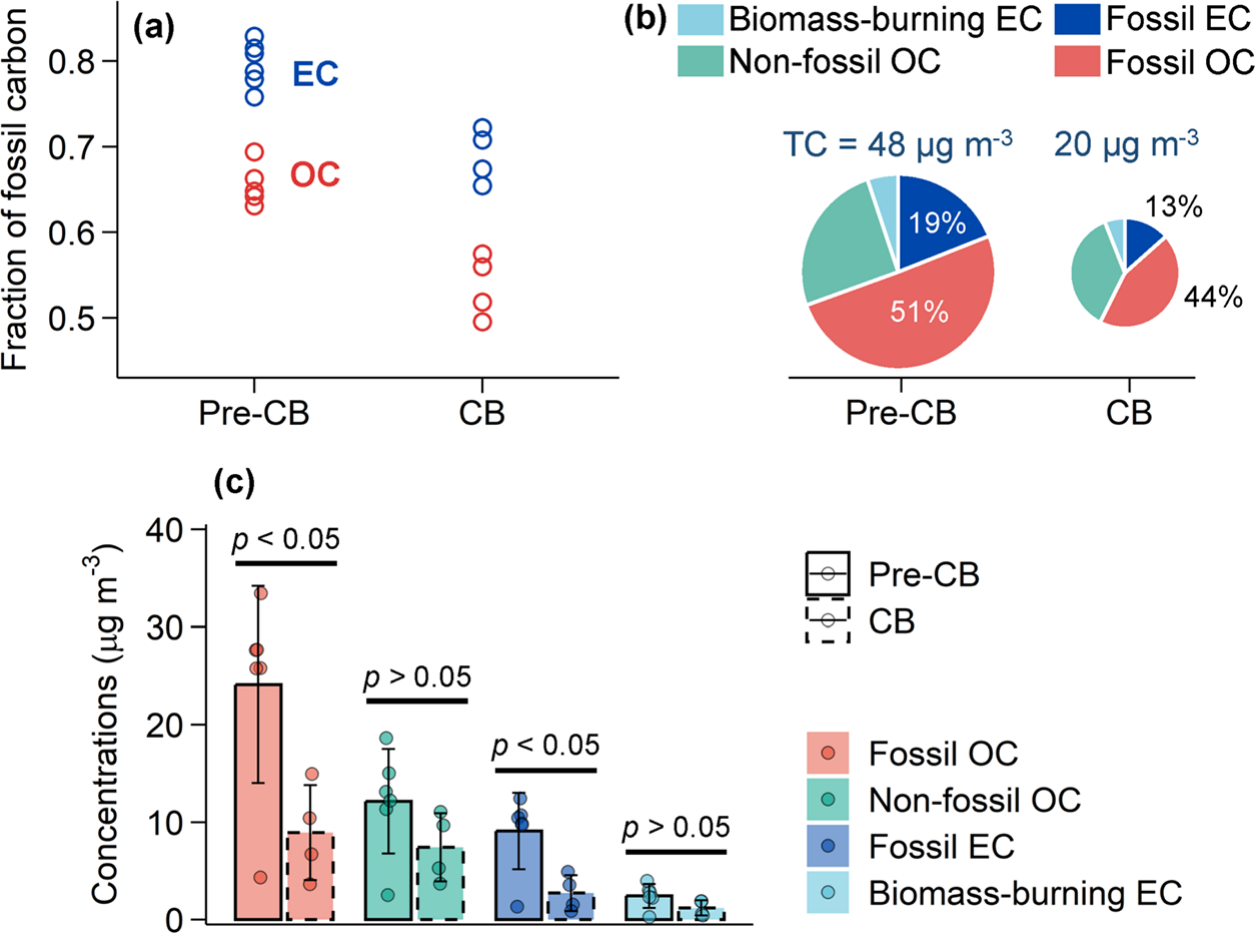
(a) Fraction of fossil carbon in elemental carbon (EC) and organic carbon (OC) during the pre-CB and CB periods; (b) average ^14^C source apportionment results of EC and OC in each period: fossil EC, biomass-burning EC (the predominant nonfossil EC), fossil OC, and nonfossil OC (e.g., OC from biomass burning, biogenic emissions, and cooking). The numbers above the pie chart are the average TC (= OC + EC) concentrations; (c) concentrations of OC and EC of fossil and nonfossil origins during the pre-CB and CB periods. The *p* values determined by *t* tests at 95% confidence level are shown in panel (c).

Radiocarbon analysis provides a reliable and highly precise method to unambiguously distinguish fossil versus nonfossil source contribution to aerosol carbon. For EC (the mass-based analogue of black carbon), the main nonfossil source is biomass burning and the fossil sources are coal and liquid fossil-fuel combustion (e.g., gasoline and diesel). During the CB period, the contribution of fossil-fuel combustion to EC was 69 ± 6% (±SD) with a range of 65–72% (Figure 3a), suggesting that even during the CB fossil sources contributed the majority of EC in Beijing. However, even larger fossil source contributions to EC were found during the pre-CB (79 ± 2%; range: 76–83%) before the CB measures. The decreased fossil fraction in EC during the CB was associated with lower EC_fossil_ concentrations compared to the pre-CB, whereas EC_bb_ concentrations did not differ significantly between CB and pre-CB periods (*p* > 0.05; Figure 3c). This provides direct ^14^C evidence of a reduction in primary fossil emissions, largely due to the reduced coal combustion. Similarly, fossil source contributions to OC (i.e., the mass of carbon items in OA) during the CB (55 ± 4%; 50–57%) were also lower than those during the pre-CB (67 ± 3%; 63–69%). As shown in Figure 3b, fossil-derived OC and EC dominated TC, with a reduced contribution during the CB than during the pre-CB (56 ± 4% vs 69 ± 2%).

**Figure 4:**
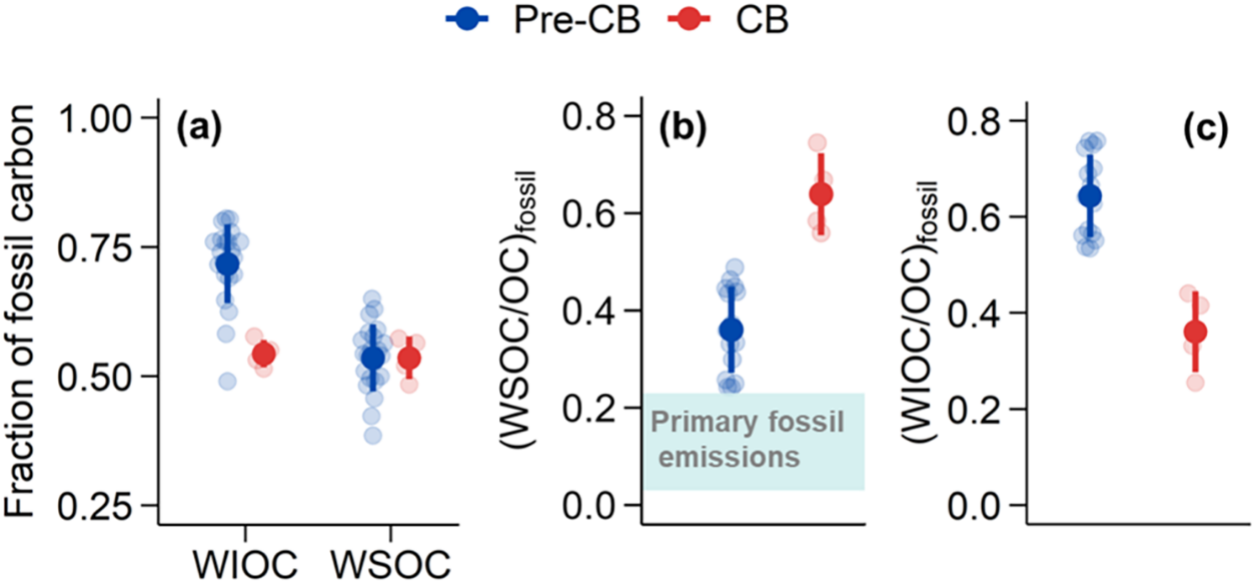
Fraction of fossil carbon in WIOC and WSOC (a) as well as (WSOC/OC)_fossil_ (b) and (WIOC/OC)_fossil_ ratios (c) during the pre-CB and CB periods in urban Beijing based on data from this study and previous studies in the literature.^66,72−74^ Error bars show the standard deviation of the mean (filled circle). Data categorized by studies are presented in Figure S1. In panel (b), the horizontal dashed area represents the characterized WSOC/OC ratios for primary fossil emissions from vehicles and coal combustion.^64−66^

The reduced fossil source contribution to OC during the CB was mainly attributed to the water-insoluble fraction of OC (WIOC), not to water-soluble OC (WSOC). The fossil fraction in WIOC (*f*_fossil_(WIOC)) decreased from 72 ± 8% (pre-CB) to 55 ± 3% (CB); however, the fossil fraction in WSOC does not change significantly (54 ± 7% vs 54 ± 4%; Figure 4). That is, the reduced coal combustion during the CB period reduced the fossil contribution to WIOC, but not the fossil contribution to WSOC. The decrease in fossil WIOC during the CB period aligns with expectations, since fossil sources typically emit large amounts of primary WIOC,^64−66^ and reduced coal combustion would reduce fossil WIOC emissions. However, it is a surprising result that the fossil contribution to the WSOC did not show a corresponding reduction. Since fossil sources generally emit only small amounts of primary WSOC,^35,64−66^ fossil WSOC being primarily of secondary origin.^67−69^ Because fossil SOA precursors (such as VOCs from coal combustion) were also likely reduced during the CB period, we would expect a decrease in fossil SOA compared to nonfossil SOA. Therefore, the observation that *f*_fossil_(WSOC) did not decrease but remained unchanged shows that during the CB period either (i) fossil SOA production was enhanced relative to contemporary SOA production or that (ii) the produced fossil SOA was more water-soluble or that (iii) photochemical aging of fossil primary OA and associated WSOC production was enhanced. Since it is unlikely that enhanced SOA production would increase fossil SOA much more strongly than nonfossil SOA, explanations (ii) or (iii) are more likely. Photochemical aging of primary OA makes it more water-soluble because OA becomes more oxidized and hygroscopic with aging.^17,70^ This effect is more important for fossil primary OA than for nonfossil primary OA because freshly emitted nonfossil primary OA is already much more water-soluble than fossil primary OA,^35,71^ and thus further oxidation has a relatively smaller effect. As a result, fossil primary OA undergoes a more significant increase in water solubility during photochemical aging compared to nonfossil primary OA, despite the likely differences in the relative aging rates of fossil and nonfossil primary OA. Another way of looking at this is by the ratios of fossil WSOC to fossil OC (abbreviated as (WSOC/OC)_fossil_; Figure 4b). Primary fossil sources including coal combustion and vehicles emit only a small fraction of WSOC (<10%),^35,64−66^ and as a consequence, primary (WSOC/OC)_fossil_ ratios are on the order of 0.05–0.2. The observed increase in (WSOC/OC)_fossil_ ratios from the pre-CB to the CB periods, resulting from a more strongly decreased fossil WIOC concentrations than fossil WSOC (Figure S15), confirms the enhanced secondary OC formation during the CB period. This conclusion from ^14^C analysis is consistent with the ACSM-PMF results discussed in Sections 3.1 and 3.2. Furthermore, the ratios of fossil WIOC to fossil OC (i.e., (WIOC/OC)_fossil_) strongly decreased from the pre-CB to the CB periods (Figure 4c). This might support the hypothesis (iii) that the increased oxidant concentrations (O_*x*_, Figure 2) during the CB period also promote the aging of fossil primary emissions and thereby contribute to enhanced fossil WSOC concentrations, despite the reduction in fossil emissions of primary OA and SOA precursors. In contrast, during the pre-CB period, the overall fossil emissions were higher, but the lower O_*x*_ levels limited the extent of photochemical aging and thus constrained the production of fossil WSOC.

### 3.4 Changes in Fossil and Nonfossil Source Contributions to Secondary OC

ACSM-PMF results reveal that the OOA was the dominating factor in total OA, especially during the CB period (Section 3.1). However, ACSM-PMF alone cannot constrain sources of OOA; in other words, initial precursors of OOA remain unidentified and unquantified at this stage. Regarding this issue, we combine the ACSM-PMF results with the ^14^C analysis to further constrain the relative importance of fossil vs nonfossil sources to OOA. To achieve this goal, the OA concentrations from the ACSM-PMF were converted to OC by dividing the characteristic OA/OC mass ratios for each OA factor (see details in Section 2.6). The sources of OC are used to explain OA sources, as OC significantly dominates the mass of OA. This consequently apportions the OOC (i.e., the mass of carbon items in OOA) into OOC of fossil origin (OOC_fossil_) and OOC of nonfossil origin (OOC_nf_), respectively (Section 2.6). In this study, we opted to compare the fractional contributions rather than the absolute mass concentrations to eliminate the uncertainties associated with mass measurement in either the ACSM or ^14^C.

**Figure 5:**
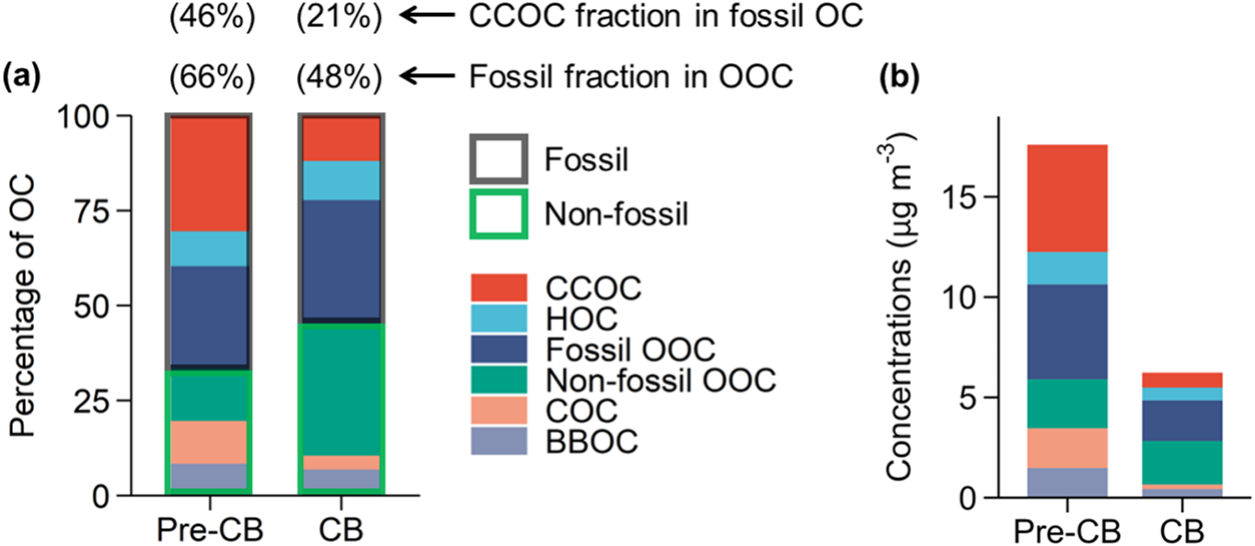
Comparison of OC from different sources between the pre-CB and CB periods. (a) Percentage contributions and (b) mass concentrations of OC factors, derived from a combination of ^14^C analysis and ACSM-PMF results. The ^14^C analysis of OC determines fossil versus nonfossil contribution to total OC with high precision. CCOC: coal combustion OC; HOC: hydrocarbon-like OC from vehicles; Fossil OOC: oxygenated OC (OOC) of fossil origin; Nonfossil OOC: nonfossil oxygenated OC; COC: cooking OC; BBOC: biomass-burning OC. In panel (a), the numbers above the bars are the percentage of the fossil fraction in the OOC and the CCOC fraction in the fossil OC (i.e., the sum of CCOC, HOC, and fossil OOC). The uncertainties (RSD) of fossil and nonfossil OOC contributions to OC are on average 14 and 18%, respectively.

As shown in Figure 5, the OOC chemical composition was significantly different between the pre-CB and CB periods. Fossil OOC accounts for 66% of OOC mass during the pre-CB period, consistent with the large emissions of OOC precursors from fossil sources (e.g., traffic emissions, coal combustion) before the implementation of coal ban measures. The large coal use for heating/cooking during the pre-CB period was verified by the larger contribution of CCOC to total OC mass (30% pre-CB vs 12% CB) and by the larger contribution of CCOC to fossil OC mass (46% pre-CB vs 21% CB). The combined results show that the fossil fraction of OOC decreased from 66% (pre-CB) to 48% (CB), attributed to the reduced amounts of precursors from coal-combustion emissions during the implementation of CB measures. In Figure 5, it can be seen that the OOC fraction of both nonfossil OOC and fossil OOC increased during the CB period, with fossil OOC fraction increasing to a lesser extent, reflecting increased oxidant concentrations and enhanced secondary formation, as discussed above. To further highlight the enhanced secondary formation during the CB period, we quantified the fossil and nonfossil OOC/POC ratios separately. The fossil-derived OOC/POC ratios increased from 0.7 (pre-CB) to 1.5 (CB), while the nonfossil-derived OOC/POC ratios increased more strongly from 0.7 (pre-CB) to 3.3 (CB). Since it is unlikely that enhanced SOA production would increase nonfossil SOA much more strongly than fossil SOA, the relatively smaller increase in the fossil-derived OOC/POC ratio and fossil OOC fraction relative to their nonfossil counterparts is attributed to the reduction in fossil OOC precursors (e.g., VOCs from coal combustion) during the CB period. It is important to note that nonfossil sources, such as biomass burning and cooking, were not directly affected by the coal ban policy. Due to the low temperatures in winter in Beijing, biogenic emissions probably produce negligible fraction of nonfossil OOA.

**Figure 6:**
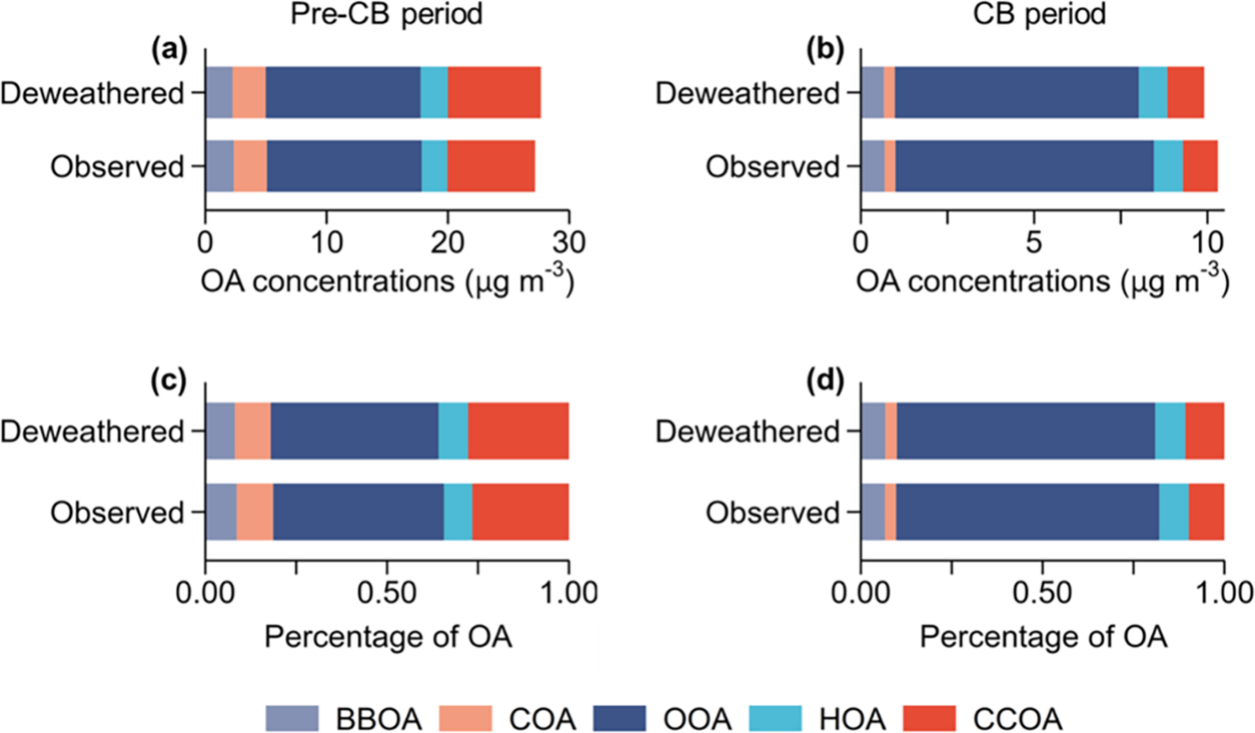
Observed and deweathered OA factors in term of concentrations and fractions in total OA mass during the pre-CB period (a, c) and CB period (b, d).

In general, stringent controls on coal combustion addressed the emission reduction of both primary particulate emissions and secondary aerosol precursors, resulting in a reduction in both the CCOC fraction in fossil OC and the fossil OOC fraction in the OOC mass. However, weather conditions (including meteorological conditions and air mass clusters) can potentially influence the variations of pollutant mass concentrations and consequently impact fractional contributions. To assess the influence of weather conditions, we compared observed concentrations during the pre-CB and CB periods with the corresponding deweathered concentrations by a machine-learning technique (Supporting Text S5), which decouples the impact of meteorology. As shown in Figure 6, the observed concentrations of OA factors were slightly different from the corresponding deweathered concentrations. Nonetheless, the changes in the fractional contributions (i.e., the mass fractions of OA factors in total OA) were minor between the observed and deweathered results. This suggests that the weather conditions had only a limited impact and justify our comparison using observed results of both the absolute and relative abundance of OA factors. Furthermore, considering that fossil WSOC is largely of secondary origin, the ^14^C-determined (WSOC/OC)_fossil_ ratios (Figure 4b) were comparable to the ratios of fossil OOC to fossil OC (i.e., (OOC/OC)_fossil_) by the combination of ACSM-PMF and ^14^C results for both per-CB and CB periods (Figure 5a; (OOC/OC)_fossil_ = 0.4 and 0.6, respectively), which increased our confidence in both methods.

## Supporting Information Available

Isolation procedures of aerosol carbon fractions for radiocarbon analysis; source apportionment method of OA resolved from ACSM using factor analysis; equations for ^14^C-based source apportionment; ^14^C-determined fossil contribution to aerosol carbon in wintertime Beijing; weather normalization technique to decouple the effects of meteorological conditions on variations in concentrations of OA factors; OA factors as a function of RH and O_*x*_ concentrations; and comparison of gas species concentrations and surface net solar radiation between the pre-CB and CB periods (PDF)
