# Supplementary material for: Reduction in Organic Aerosol from Coal Combustion is Partially Offset by Enhanced Secondary Formation during the Beijing Coal Burning Ban
Source: Environ Sci Technol. 2025 May 5;59(18):9155–66. doi: 10.1021/acs.est.4c13051 (PMC12080253; doi:10.1021/acs.est.4c13051)
Supplement: Supplementary file 1 — es4c13051_si_001.pdf [file es4c13051_si_001.pdf]

Supporting Information for

Reduction in organic aerosol from coal combustion is partially offset by enhanced secondary formation during the Beijing coal burning ban

Haiyan Ni, Haobin Zhong, Ying Wang, Peng Yao, Jie Tian, Yongyong Ma, Ru-Jin Huang, Ulrike Dusek

Correspondence: Ulrike Dusek (u.dusek@rug.nl); Ru-Jin Huang (huang@ieecas.cn)

This supporting material includes:

21 pages,

6 Supplement sections,

15 Figures and 3 Tables.

Text S1. Isolation of carbon fractions for radiocarbon analysis

The separation of OC, WIOC and EC was performed on our custom-built aerosol combustion system,[1] where aerosol samples are combusted in pure O_2. To isolate OC, aerosol samples were heated at 375°C for 10 min. Before extraction of WIOC and EC, water-soluble OC (WSOC) is removed from filter pieces by water extraction to minimize the formation of charred OC.[2] Then, the water-extracted filter piece was heated at 375°C for 10 min to extract WIOC. To completely remove OC before EC extraction, the oven temperature is then increased to 450°C for 3 min to remove the most refractory OC that is left on the filters. Finally, EC is isolated by heating the remaining carbon at 650°C for 5 min. Detailed isolation procedures can be found in Dusek et al.[3] and Zenker et al.[4]

Text S2. Source apportionment of OA using PMF and ME-2

In this study, Positive Matrix Factorization (PMF) was used to perform source apportionment of organic mass spectra and time series in a Time-of-Flight Aerosol Chemical Speciation Monitor (ToF-ACSM), which has been widely utilized in source apportionment studies of organic matter.[5-7] The overall objective of the source analysis was to quantitatively apportion the sources of organic aerosols (OA) measured by the ToF-ACSM.

PMF is a bilinear receptor model capable of separating the input data matrix into distinct factor matrices and their corresponding time series of concentrations.[8,9] It describes as follows:

$$X = GF + E \tag{S1}$$

where X represents the original matrix ($i \times j$) of the time series (i) and mass spectrum (j) of organics measured by ToF-ACSM. In other words, the i denotes rows of the observed matrix X (i = 1, …, n) and j denotes columns of X (j = 1, …, m). F and G denote the factor matrix ($p \times j$) of the mass spectrum (j) of organics under different factor numbers (p) and the weight matrix ($i \times p$) of the time series of organics (i) for each factor, respectively. E indicates the residual between the reconstructed matrix, calculated by multiplying F and G, and the original matrix X. To reduce the residual, a weighted sum of squared residuals was introduced in the calculations, as described below:

$$Q = \sum_{i=1}^{n}\sum_{j=1}^{m}\left(\frac{e_{ij}}{\sigma_{ij}}\right)^2 \tag{S2}$$

where Q represents the sum of squared residuals after weighting, which is the ratio of the residual of the jth species (e_{ij}) in the ith hour in the matrix to its standard deviation (σ_{ij}).

The results of PMF revealed five sources of organic matter, namely HOA, BBOA, CCOA,

COA, and OOA. HOA (hydrocarbon-like OA) is characterized by alkyl fragment ion series, which are in the form of C_nH_{2n-1} and C_nH_{2n+1}, and has marker ions such as *m/z* 41, 43, 55, 57, 69, 71, 83 and 85 in the spectra.[10,11] BBOA (biomass burning OA) is identified by prominent peaks at *m/z* 60 ($C_2H_4O_2^+$) and *m/z* 73 ($C_3H_5O_2^+$).[12,13] These are fragments of levoglucosan and mannosan which are known pyrolysis products of incomplete biomass burning.[14] CCOA (coal combustion OA) correlates highly with PAH-related ion fragments, including *m/z* 77, 91, 105 and 115, and is also characterized by unsaturated fragments at higher *m/z*.[15,16] COA (cooking OA) has a similar spectrum to HOA but with higher signal at *m/z* 41 and *m/z* 55; thus, the ratio of *m/z* 41/43 and *m/z* 55/57 is typically used to identify its profile.[17,18] OOA is distinguished by prominent ions at *m/z* 44 (CO_2^+) and *m/z* 43 ($C_2H_3O^+$), and fewer signals at higher *m/z* owing to high oxidation.[19]

To further reduce mixing between factors, the Multilinear Engine 2 (ME-2) was utilized as a solution for PMF.[9] This PMF-ME-2 approach has been effectively utilized in previous source apportionment studies of aerosol mass spectrometry data.[20-22] ME-2 constrains the F matrix in PMF using known online mass spectral profiles of OA, reducing the randomness and uncertainty introduced by the double unknown matrices in the PMF model. By constraining certain factors in the F matrix to fixed mass spectral profiles, ME-2 reduces rotational ambiguity inherent in the base PMF model and provides a solution more chemically reasonable and stable.

HOA was constrained using the profile from Ng et al.,[23] which represented the average of 15 sites globally including East Asia, Europe and North America. A previous study discussed the similarity of HOA profiles from the United States (gasoline-dominated) and Europe (diesel-dominated) using cosine similarity analyses, suggesting HOA profiles from different vehicle types were nearly equivalent.[24] For HOA constraints, different a values ranging from 0 to 1 (with a step of 0.1) were tested based on results of the 5-factor PMF solutions. The final HOA results were obtained by averaging solutions with a values from 0 to 0.9, as this range showed stable performance with lower $Q/Qexp$ values compared to unconstrained PMF ($a = 1$; Fig. S3). CCOA and COA profiles were fully constrained ($a = 0$) from Elser et al.[24] and Duan et al.[25], respectively, as these profiles were obtained from the same sampling location. BBOA and OOA factors were left unconstrained in the analysis. The sensitivity test results for HOA are presented in Figures S4–S7. Figure S4 presents the $Q/Qexp$ ratios under different a-values, showing that solutions with a values from 0 to 0.9 yielded lower $Q/Qexp$ values compared to the unconstrained case ($a = 1$). Figure S5 displays the mass spectra profiles, where the error

bars represent the standard deviation of mass spectral variations across different *a* values, showing 3%–23% variations in unit mass resolution signals. Figure S6 shows the residual analysis results, indicating stable model performance across the tested *a* value range. Figure S7 presents the time series comparison, demonstrating consistent temporal patterns with approximately 5% uncertainty. These results confirm that averaging solutions with *a* values from 0 to 0.9 provides optimal and stable performance in terms of both mass spectral features and temporal variations.

Values of HOA, COA and CCOA constraints ranging from 0 to 1 with a step of 0.1 were tested based on results of the 5-factor PMF solutions. The outcomes are averaged and depicted in Figure S2. The time series of both the constrained and unconstrained factors were highly correlated with corresponding external data. The coefficient of determination, denoted as R^2, between HOA, BBOA, CCOA, COA, and OOA with black carbon, $C_2H_4O_2^+$, chloride, $C_6H_{10}O^+$, and secondary inorganic aerosol (SO_4^{2-} + NO_3^- + NH_4^+) were 0.43, 0.79, 0.61, 0.66, and 0.78, respectively, validating the accuracy of the source apportionment results. All calculations related to ME-2 were performed on SourceFinder (6.3en, Datalystica, Ltd., Park InnovAARE, Villigen, Switzerland)[26] embedded in Igor Pro software (v6.37, WaveMetrics, Inc., Lake Oswego, OR, USA).

Text S3. The technical comparability of ACSM measurements between winters 2015/16 and 2017/18

The technical comparability of ACSM measurements between winter 2015/16 (pre-CB period) and winter 2017/18 (CB period) was ensured by using consistent instrumental configurations. Both ACSM measurements were conducted using standard vaporizers (SV) with collection efficiency (CE) corrections. The CE values were determined through comparisons with concurrent scanning mobility particle sizer (SMPS) measurements for the 2015/16 campaign and with $PM_{2.5}$ data from the nearest China National Environmental Monitoring Center (CNEMC) station for the 2017/18 campaign. The consistent use of SV and CE correction methods across both periods ensures the reliability of comparing ACSM data from different measurement campaigns, despite different particle size cuts ($PM_{1.0}$ in 2015/16 and $PM_{2.5}$ in 2017/18; Sect. 2.5).

Text S4. ^{14}C-determined fossil contribution to aerosol carbon in wintertime Beijing in the literature

The ^{14}C-determined fraction of fossil carbon in EC, OC, WIOC and WSOC (f_{fossil}(EC),

f_{fossil}(OC), f_{fossil}(WIOC) and f_{fossil}(WSOC), respectively) in winter 2017/18 during the implementation of coal ban (CB) policy (referred as to the CB period) as well as previous winters (referred as to the pre-CB period) in urban Beijing are presented in Figure S1 and Table S1 based on our ^{14}C results and data from previous studies.[27-33] The f_{fossil}(EC), f_{fossil}(OC) and f_{fossil}(WIOC) was much smaller during the pre-CB period, compared to the pre-CB periods. In contrast, f_{fossil}(WSOC) did not change significantly between the pre-CB and CB periods (see detailed discussion in Sect. 3.3).

In the winters before the implementation of CB (refer to as pre-CB periods), the f_{fossil}(EC), f_{fossil}(OC), f_{fossil}(WSOC) and f_{fossil}(WIOC) from the literature show relatively stable values over the pre-CB winters, except for f_{fossil}(WSOC) data from Zhang et al,[28] which is an outlier compared to the other studies. Excluding the Zhang et al.[28] data does not significantly alter the average pre-CB f_{fossil}(WSOC) of 56% ± 5% compared to the value obtained when including these data (54% ± 7%). This increases our confidence to combine the previous ^{14}C data in the literature for a pre-CB ^{14}C estimate. When combining the ^{14}C data of OC with the ACSM data (see Sect. 2.6), we notice that for the pre-CB period, most of the ^{14}C data were from December−January while the ACSM data were from February−March, that is, the ^{14}C and ACSM data were from different winter months. However, available Beijing ^{14}C data shows limited ^{14}C variability between these winter months. For example, our f_{fossil}(OC) was 57% ±1 % in December-January, similar to 52% ± 3% in February-March in urban Beijing (Table S1). Another evidence comes from Kirillova et al.[34], who reported a f_{fossil}(OC) of 59% ± 4% at a receptor station during 8−13 March 2011 for air masses originating from the Beijing area, consistent with December-January f_{fossil}(OC) values in the pre-CB winters in urban Beijing (Table S1). In other words, f_{fossil}(OC) values do not vary strongly across winter months. This justifies the combination of ^{14}C data of OC with the ACSM data from different winter months.

Text S5. Weather normalization technique

A machine learning-based random forest (RF) algorithm model, in combination with the OA source apportionment results, was employed to decouple the effects of meteorological conditions on variations in air pollutant concentrations, helping the comparison of the pollutant emission levels.[35,36] Time variables, meteorological data, air mass clusters were used as input predictor features to build RF model (Sect. 2.7). The parameter setup for RF model was the same with Vu et al.[35] as follows: tree numbers of 300, minimal node size of 3, the number of variables split at each node of 3. Model performance of OA factors was good, proved by model

performance parameters, including Pearson's R-value (> 0.85), root mean square error (2.5–5.5), fraction of predictions with a factor of two (0.6–0.8), mean bias (-0.1–0.2), mean gross error (1.5–3.2), normalized mean bias (-0.01–0.02), normalized mean gross error (0.29–0.38), coefficient of efficiency (0.5–0.6), and index of agreement (0.75–0.80) for the per-CB period, as detailed in our previous study.[37] For the CB period, the model performance parameters for each OA factors are listed in Table S3. Conducted using the "rmweather" R package (available at https://cran.r-project.org/web/packages/rmweather/index.html), the weather normalization resampled the weather variables from the whole study period and randomly allocated to a dependent variable observation. The selection process was repeated 1000 times to gain 1000 predicted concentrations of each OA factor. The deweathered concentrations of each OA factor were obtained by averaging the 1000 predicted values.

Text S6. Changes in concentrations of gas species

SO_2, CO and NO_x: Figure S9 shows the concentrations of SO_2, CO and NO_x during the pre-CB and CB periods that contemporaneous with our ACSM sampling period. Ground-level concentrations of SO_2 and CO were obtained from the Beijing Municipal Ecological and Environmental Monitoring Center (https://www.bjmemc.com.cn/). NO_x concentrations were directly measured using a NO_x analyzer (Thermo Fisher Scientific, Model 42i) during our sampling campaign.

SO_2 concentrations decreased significantly from 20.7 μg m^{-3} during the pre-CB period to 10.5 μg m^{-3} during the CB period (Fig. S9). This reduction aligns with our expectations, as coal combustion is the primary source of SO_2 in China. The mean concentrations of CO also decreased during the CB period (though there is no significant difference at the 95% confidence level, $p > 0.05$), but the reduction in CO levels was less significant compared to that of SO_2. This is related to more diverse sources of CO, including biomass burning and vehicular emissions that were not directly affected by the coal ban policy. In addition, the combustion of natural gas also emits CO, but does not emit SO_2. The decreased SO_2 and CO concentrations during the implementation of coal ban policy were also observed in previous studies.[38,39]

Unlike SO_2 and CO, the observed NO_x concentrations increased during the CB period, compared to the pre-CB period ($p < 0.05$). The coal ban policy involves replacing coal with natural gas for heating (the "coal to gas" transition). NO_x emissions from combustion of natural gas are higher than from coal combustion due to higher combustion temperatures. Higher combustion temperatures strongly increase thermal NO_x emissions, which are caused by the

thermal dissociation and subsequent reaction of N_2 and O_2 molecules in the combustion air. Vehicular emissions are another major source of NO_x, but the coal ban policy does not directly impact vehicle emissions. Our observation indicates that the additional NO_x emissions from natural gas emissions during the “coal to gas” transition surpassed the NO_x reduction from the decreased coal combustion. Our findings align with those of Zhao et al. (2020),[39] who reported a similar rise in NO_x concentrations (and a significant decrease in SO_2) during the winter with the "coal to gas" transition in the greater Beijing-Hebei-Tianjin region.

O_x concentrations: The changes in concentrations of O_x, the sum of O_3 and NO_2 that were measured using standard gas analyzers (Thermo Fisher Scientific, Model 49i and 42i, respectively), as well as the influence of O_x on OOA, are discussed in Sect. 3.2 of the main text.

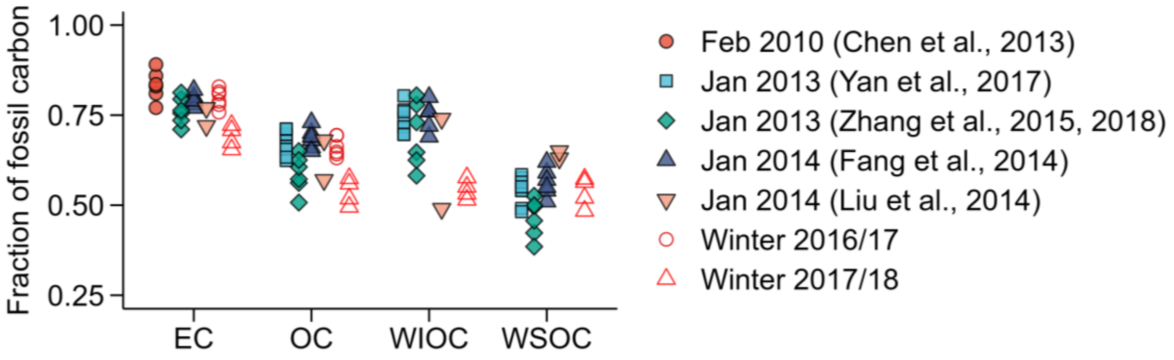

Figure S1. ^{14}C-determined fraction of fossil carbon in EC, OC, WIOC and WSOC (f_{fossil}(EC), f_{fossil}(OC), f_{fossil}(WIOC), f_{fossil}(WSOC)) in wintertime Beijing based on our ^{14}C results and data from previous studies.[27-33]

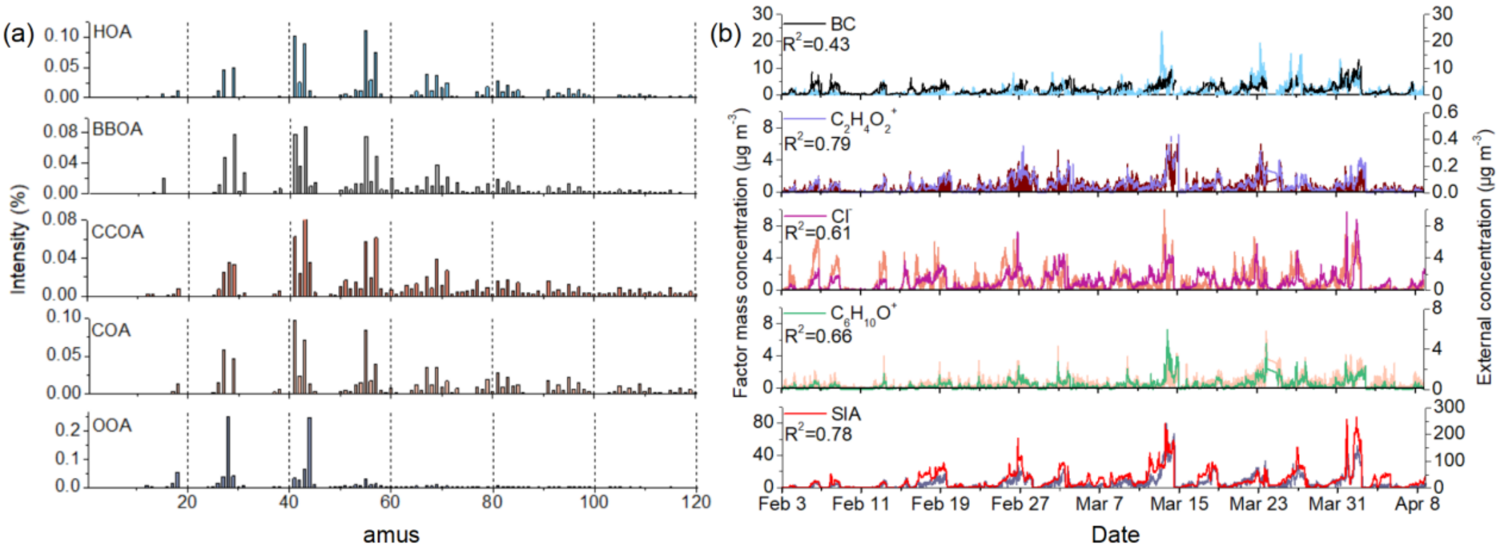

Figure S2. (a) Optimization of PMF factor constraints in the ME-2 analysis and the **(b)** time series of constrained factors with their corresponding external tracers. R^2 represents the coefficient of determination between the constrained factors' time series and the corresponding external tracers. In panel b, the R^2 between HOA and black carbon, BBOA and $C_2H_4O_2^+$, CCOA and Cl^-, COA and $C_6H_{10}O^+$, and OOA and SIA ($SO_4^{2-} + NO_3^- + NH_4^+$) were 0.43, 0.79, 0.61, 0.66, and 0.78, respectively.

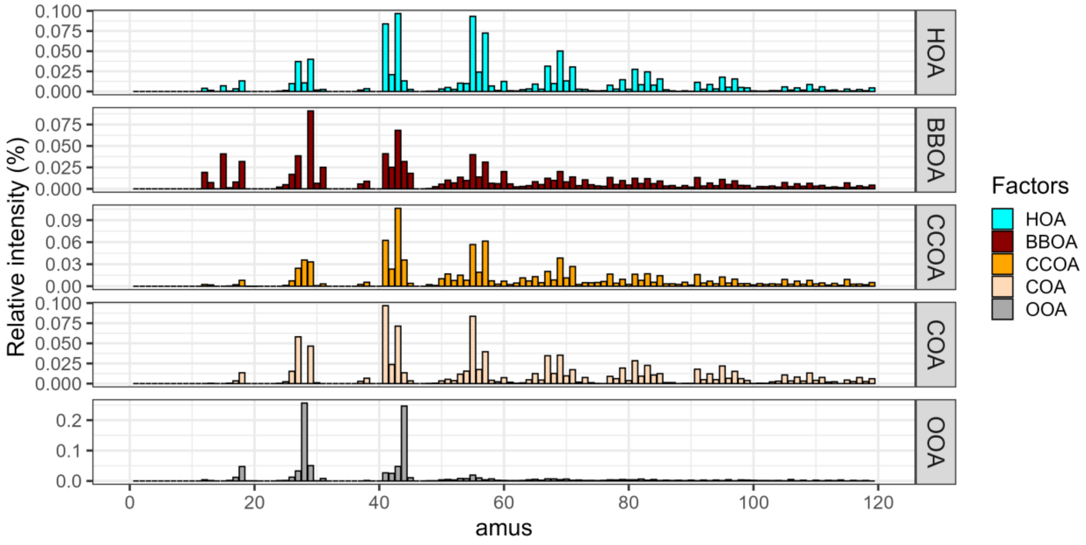

Figure S3. Mass spectral profiles of 5 factors resolved from unconstrained PMF analysis.

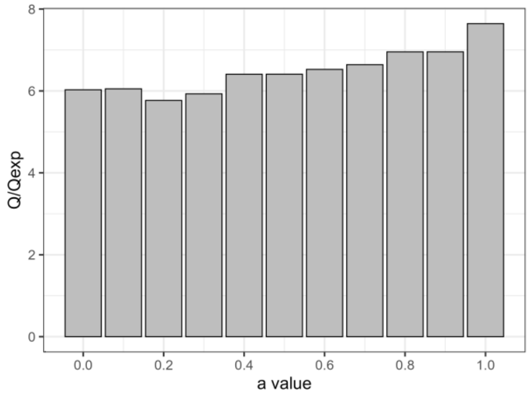

Figure S4. *Q/Qexp* values as a function of different *a* values (0–1, step = 0.1) for HOA constraints in ME-2 analysis.

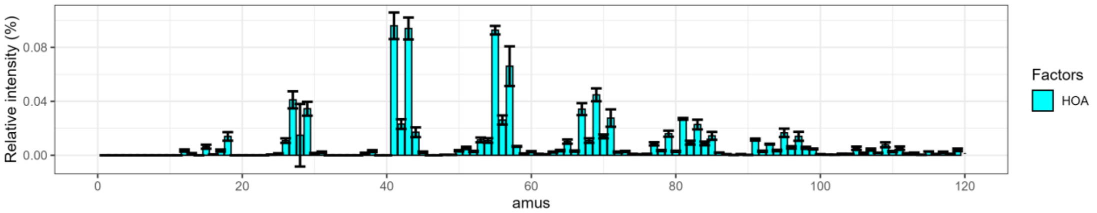

Figure S5. Average mass spectral profile of HOA factor obtained from ME-2 solutions with *a* values ranging from 0 to 0.9. Error bars represent the standard deviation of mass spectral variations across different *a* values, indicating the uncertainty introduced by varying *a* values.

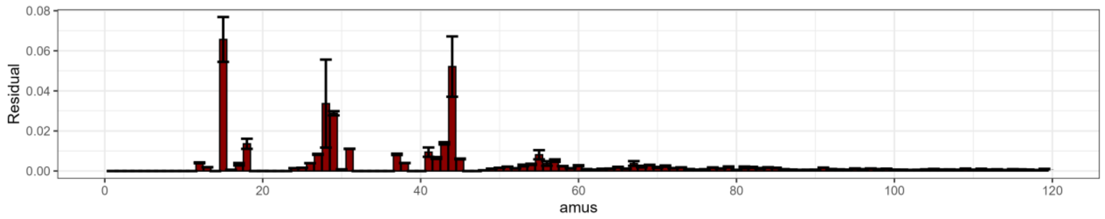

Figure S6. Average residual analysis results of HOA factor obtained from ME-2 solutions with *a* values ranging from 0 to 0.9. Error bars represent the standard deviation of residual variations across different *a* values.

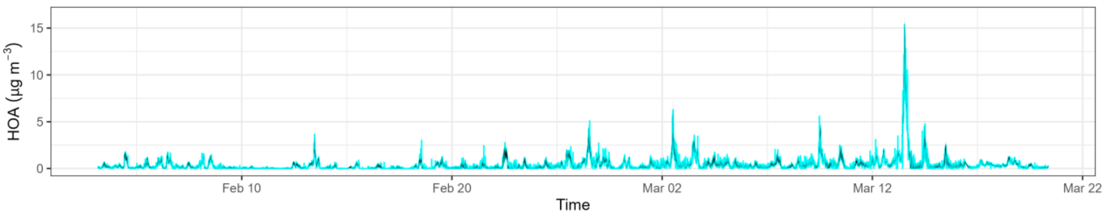

Figure S7. Average time series of HOA factor obtained from ME-2 solutions with *a* values ranging from 0 to 0.9. The shaded area (black ribbon) represents the standard deviation of temporal variations across different *a* values.

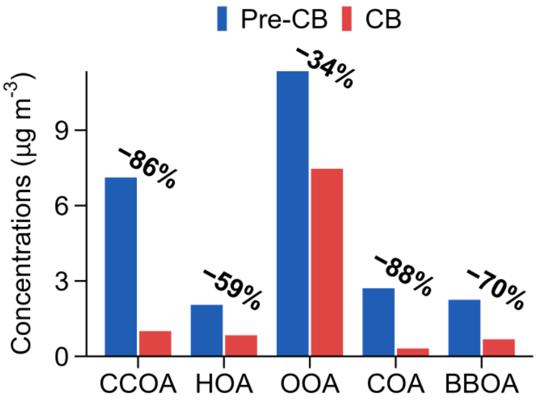

Figure S8. Concentrations of OA factors, including both primary (CCOA, HOA, COA, BBOA) and secondary species (OOA) during the pre-CB and CB period. The numbers are the percentage changes of each OA factor from the pre-CB to the CB periods.

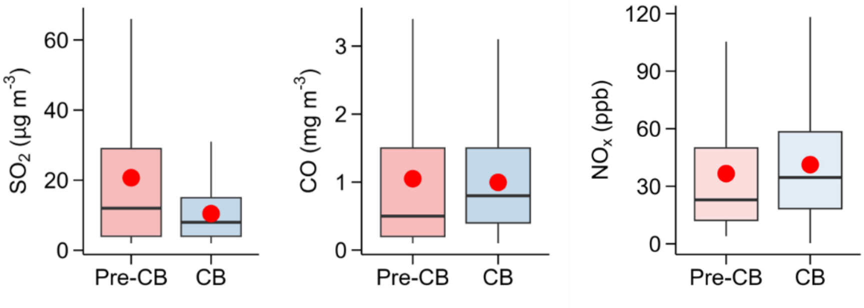

Figure S9. Concentrations of SO_2, CO and NO_x during the pre-CB and CB periods. The pre-CB and CB periods are consistent with our ACSM measurements of organic aerosol (OA). The filled circle within each box indicates the mean, the box denotes the interquartile range with the box center line representing the median. See the Text S6 for a detailed discussion on how gas species changed from pre-CB to CB period.

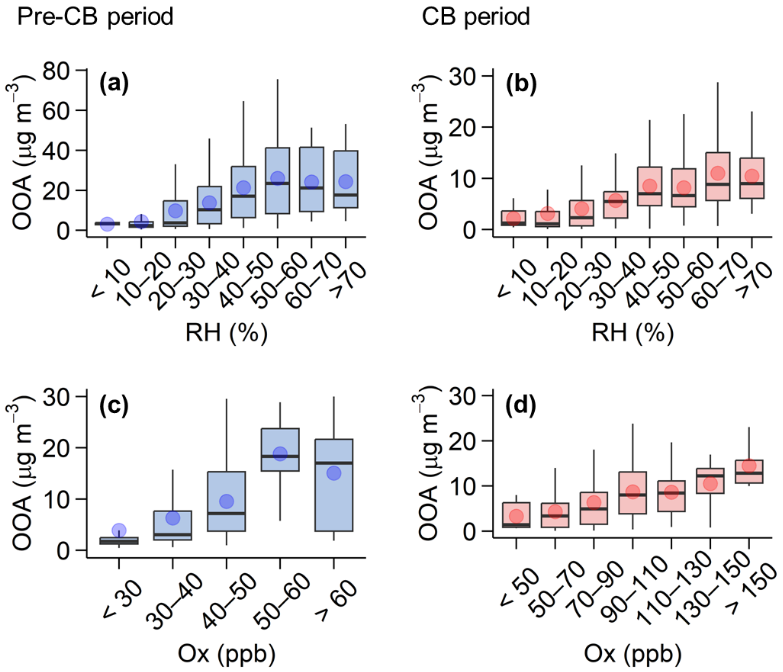

Figure S10. The OOA concentrations as functions of RH (%) and O_x concentrations (ppb) during the pre-CB **(a, c)** and CB periods **(b, d)**. In panels a–d, the filled circle within each box indicates the mean, the box denotes the interquartile range with the box center line representing the median.

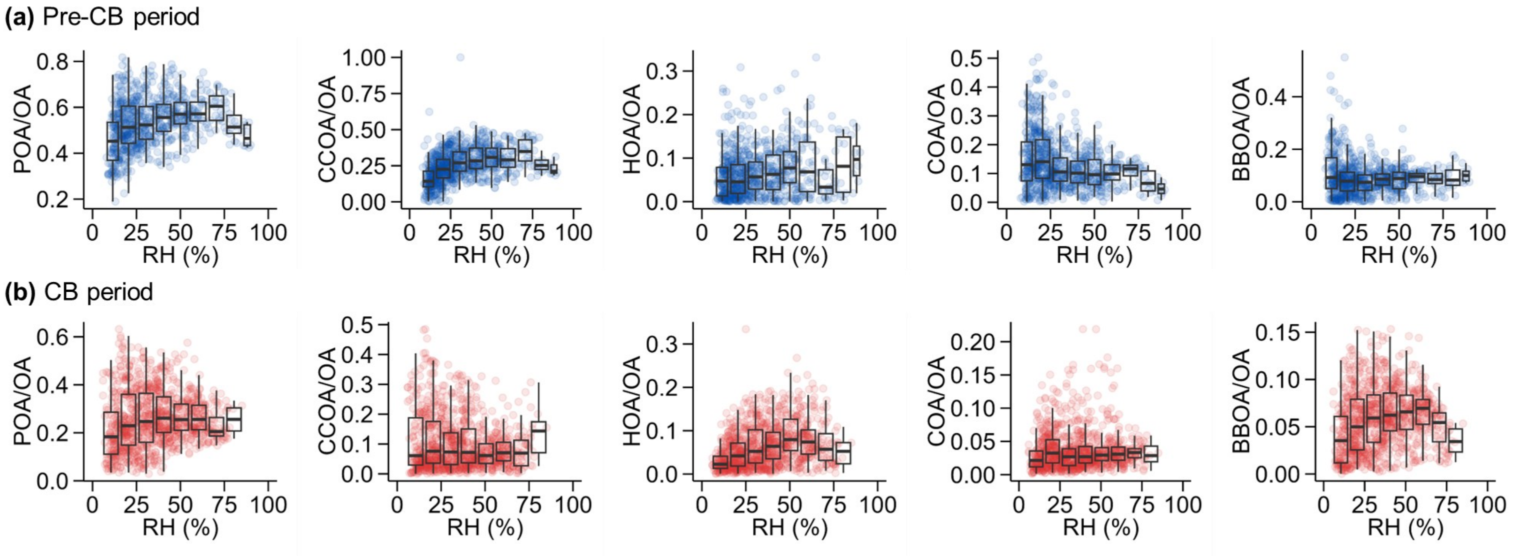

Figure S11. The mass fraction of POA factors in total OA (i.e., POA/OA) as functions of RH during the pre-CB **(a)** and CB periods **(b)**. Data were binned according to RH (ΔRH = 10%). POA factors include coal combustion OA (CCOA), hydrocarbon-like OA (HOA), cooking OA (COA) and biomass burning OA (BBOA).

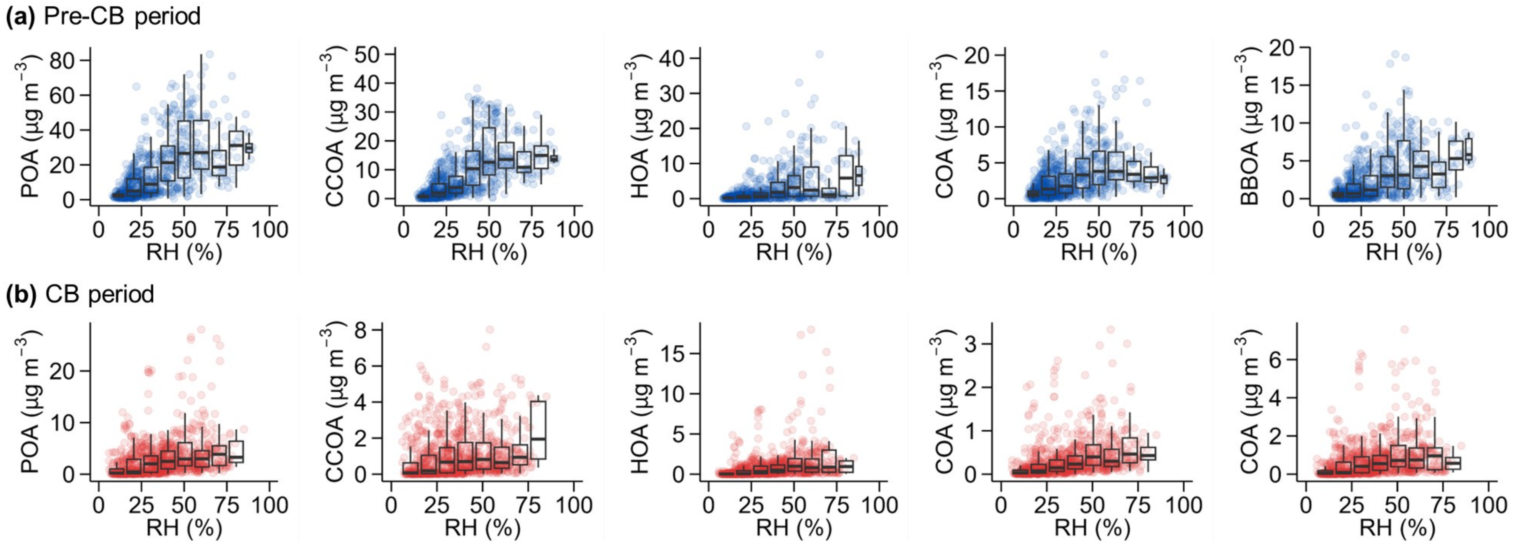

Figure S12. Concentrations of POA factors as functions of RH during the pre-CB **(a)** and CB period **(b)**. Data were binned according to RH (ΔRH = 10%). POA factors include coal combustion OA (CCOA), hydrocarbon-like OA (HOA), cooking OA (COA) and biomass burning OA (BBOA).

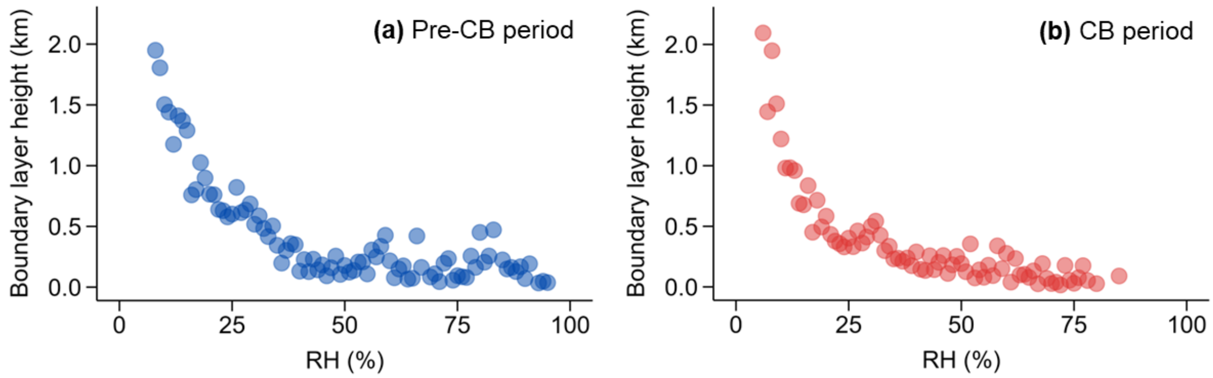

Figure S13. Correlations between boundary layer heights and RH during the pre-CB and CB periods.

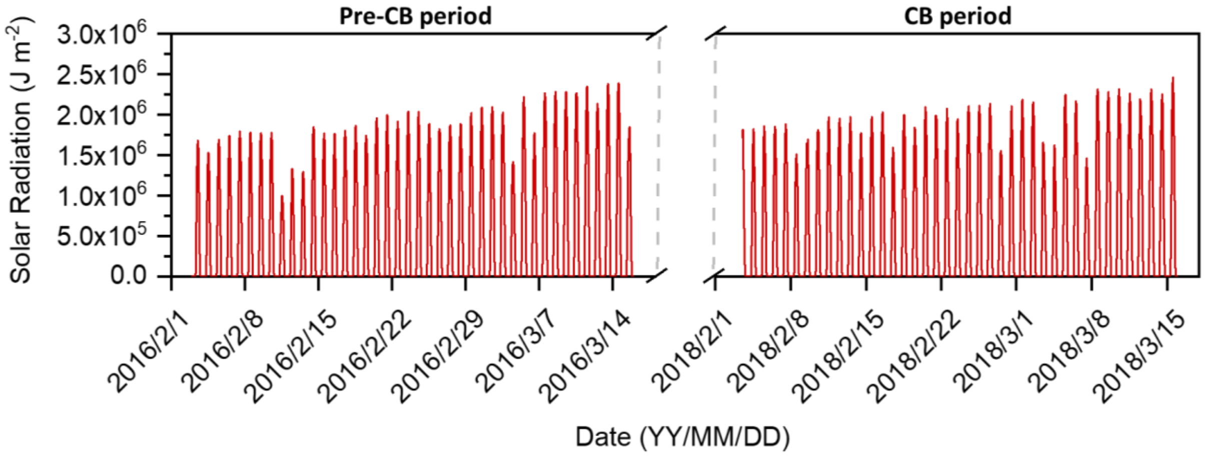

Figure S14. Time series of surface net solar radiation for the pre-CB and CB periods. Surface net solar radiation was obtained from ERA5 reanalysis dataset.

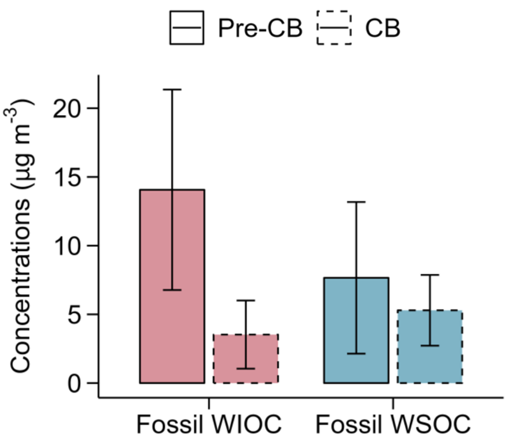

Figure S15. Concentrations of fossil-derived WIOC and WSOC during the pre-CB and CB periods.

Table S1. Selected offline filter samples for ^{14}C analysis in this study as well as the collected ^{14}C data from literature. These data are also present in Figure S1. The f_{fossil}(OC) for CB and pre-CB periods are averaged separately and then combined with the corresponding averages of ACSM-PMF results, as detailed in Sect. 2.6.

Period	Sampling date	$PM_{2.5}$ (μg m^{-3})	f_{fossil}(EC)	f_{fossil}(OC)	f_{fossil}(WIOC)	f_{fossil}(WSOC)	Source
Coal Ban (CB)	29 Dec 2017	175	0.722 ± 0.036	0.575 ± 0.006	0.577 ± 0.008	0.573 ± 0.012	This study (winter 2017/18)
	12 March 2018	142	0.655 ± 0.027	0.518 ± 0.009	0.515 ± 0.013	0.520 ± 0.017	
	2 March 2018	77	0.708 ± 0.011	0.560 ± 0.010	0.551 ± 0.016	0.564 ± 0.008	
	20 Feb 2018<br>4 March 2018<br>8 March 2018	45<br>57<br>43	0.674 ± 0.014	0.495 ± 0.017	0.532 ± 0.022	0.484 ± 0.017	
Pre-CB	11 Dec 2016	212	0.779 ± 0.006	0.648 ± 0.008			This study (winter 2016/17)[33]
	17 Dec 2016	246	0.788 ± 0.006	0.663 ± 0.008			
	20 Dec 2016	357	0.758 ± 0.006	0.642 ± 0.008			
	1 Jan 2017<br>2 Jan 2017	251<br>219	0.815 ± 0.006	0.694 ± 0.007			
	4 Jan 2017	270	0.809 ± 0.004	0.694 ± 0.007			
	26 Dec 2016<br>8 Jan 2017	16<br>12	0.829 ± 0.006	0.630 ± 0.008			
	8 January 2014		0.72	0.57	0.49	0.63	Liu et al. (2014)[32]
	15 January 2014		0.77	0.68	0.74	0.65	
	January 2014		0.793 ± 0.018	0.685 ± 0.029	0.748 ± 0.038	0.563 ± 0.039	Fang et al. (2014)[30]
	January 2013		0.762 ± 0.037	0.587 ± 0.051	0.695 ± 0.090	0.464 ± 0.053	Zhang et al. (2015, 2018)[28,29]
	January 2013		/	0.673 ± 0.034	0.737 ± 0.037	0.542 ± 0.037	Yan et al. (2017)[31]
	February 2010		0.833 ± 0.041	/	/	/	Chen et al. (2013)[27]

Table S2. Equations for ^{14}C-based source apportionment. Carbon mass from non-fossil sources (EC_{bb}, OC_{nf}, $WIOC_{nf}$, $WSOC_{nf}$) and from fossil sources (EC_{fossil}, OC_{fossil}, $WIOC_{fossil}$, $WSOC_{fossil}$) were determined using equations S3–S13. In Eq. S5, $F^{14}C_{nf}$ is $F^{14}C$ of non-fossil sources, and estimated as to be 1.10 ± 0.05 for EC and 1.09 ± 0.05 for OC fractions, as detailed in Ni et al.[40]

Equations	
$F^{14}C_{WSOC} = \frac{F^{14}C_{OC} \times OC - F^{14}C_{WIOC} \times WIOC}{OC - WIOC}$	(S3)
$WSOC = OC - WIOC$	(S4)
$f_{nf} = \frac{F^{14}C}{F^{14}C_{nf}}$	(S5)
$EC_{bb} = EC \times f_{bb}(EC)$	(S6)
$EC_{fossil} = EC \times (1 - f_{bb}(EC)) = EC \times f_{fossil}(EC)$	(S7)
$OC_{nf} = OC \times f_{nf}(OC)$	(S8)
$OC_{fossil} = OC \times (1 - f_{nf}(OC)) = OC \times f_{fossil}(OC)$	(S9)
$WIOC_{nf} = WIOC \times f_{nf}(WIOC)$	(S10)
$WIOC_{fossil} = WIOC \times (1 - f_{nf}(WIOC)) = WIOC \times f_{fossil}(WIOC)$	(S11)
$WSOC_{nf} = WSOC \times f_{nf}(WSOC)$	(S12)
$WSOC_{fossil} = WSOC \times (1 - f_{nf}(WSOC)) = WSOC \times f_{fossil}(WSOC)$	(S13)

Table S3. The RF model performance parameters for testing data set of OA factors during the CB period.

OA factors	FAC2*	MB	MGE	NMB	NMGE	RMSE	*r*	COE	IOA
BBOA	0.714	-0.0297	0.203	-0.0436	0.298	0.327	0.929	0.671	0.836
COA	0.737	0.00724	0.107	0.0231	0.342	0.166	0.909	0.601	0.8
OOA	0.884	-0.204	1.42	-0.0263	0.183	2.56	0.976	0.784	0.892
HOA	0.651	-0.0497	0.311	-0.0547	0.342	0.82	0.904	0.68	0.84
CCOA	0.608	0.00829	0.4	0.00816	0.394	0.587	0.905	0.586	0.793

*FAC2 (fraction of predictions with a factor of two), MB (mean bias), MGE (mean gross error), NMB (normalized mean bias), RMSE (root-mean-square deviation), NMGE (normalized mean gross error), *r* (correlation coefficient), COE (coefficient of efficiency), IOA (index of agreement).

REFERENCES

(1) Dusek, U.; Monaco, M.; Prokopiou, M.; Gongriep, F.; Hitzenberger, R.; Meijer, H. A. J.; Röckmann, T. Evaluation of a two-step thermal method for separating organic and elemental carbon for radiocarbon analysis. *Atmos. Meas. Tech.* **2014**, *7*, 1943–1955.

(2) Yu, J. Z.; Xu, J.; Yang, H., Charring characteristics of atmospheric organic particulate matter in thermal analysis. *Environ. Sci. Technol.* **2002**, *36*, 754–761.

(3) Dusek, U.; Hitzenberger, R.; Kasper-Giebl, A.; Kistler, M.; Meijer, H. A.; Szidat, S.; Wacker, L.; Holzinger, R.; Röckmann, T. Sources and formation mechanisms of carbonaceous aerosol at a regional background site in the Netherlands: Insights from a year-long radiocarbon study. *Atmos. Chem. Phys.* **2017**, *17*, 3233–3251.

(4) Zenker, K.; Vonwiller, M.; Szidat, S.; Calzolai, G.; Giannoni, M.; Bernardoni, V.; Jedynska, A. D.; Henzing, B.; Meijer, H. A.; Dusek, U. Evaluation and inter-comparison of oxygen-based OC-EC separation methods for radiocarbon analysis of ambient aerosol particle samples. *Atmosphere* **2017**, *8*, 226, https://doi.org/10.3390/atmos8110226.

(5) Crippa, M.; Canonaco, F.; Lanz, V. A.; Äijälä, M.; Allan, J. D.; Carbone, S.; Capes, G.; Ceburnis, D.; Dall'Osto, M.; Day, D. A.; DeCarlo, P. F.; Ehn, M.; Eriksson, A.; Freney, E.; Hildebrandt Ruiz, L.; Hillamo, R.; Jimenez, J. L.; Junninen, H.; Kiendler-Scharr, A.; Kortelainen, A. M.; Kulmala, M.; Laaksonen, A.; Mensah, A. A.; Mohr, C.; Nemitz, E.; O'Dowd, C.; Ovadnevaite, J.; Pandis, S. N.; Petäjä, T.; Poulain, L.; Saarikoski, S.; Sellegri, K.; Swietlicki, E.; Tiitta, P.; Worsnop, D. R.; Baltensperger, U.; Prévôt, A. S. H. Organic aerosol components derived from 25 AMS data sets across Europe using a consistent ME-2 based source apportionment approach. *Atmos. Chem. Phys.* **2014**, *14*, 6159–6176.

(6) Liu, D.; Allan, J.; Young, D.; Coe, H.; Beddows, D.; Fleming, Z. L.; Flynn, M.; Gallagher, M.; Harrison, R.; Lee, J.; Prevot, A. S. H.; Taylor, J. W.; Yin, J., Williams, P. I.; Zotter, P. Size distribution, mixing state and source apportionment of black carbon aerosol in London during wintertime. *Atmos. Chem. Phys.* **2014**, *14*, 10061–10084.

(7) Chen, G.; Canonaco, F.; Tobler, A.; Aas, W.; Alastuey, A.; Allan, J.; Atabakhsh, S.; Aurela, M.; Baltensperger, U.; Bougiatioti, A.; De Brito, J. F.; Ceburnis, D.; Chazeau, B.; Chebaicheb, H.; Daellenbach, K. R.; Ehn, M.; El Haddad, I.; Eleftheriadis, K.; Favez, O.; Flentje, H.; Font, A.; Fossum, K.; Freney, E.; Gini, M.; Green, D. C.; Heikkinen, L.; Herrmann, H.; Kalogridis, A.-C.; Keernik, H.; Lhotka, R.; Lin, C.; Lunder, C.; Maasikmets, M.; Manousakas, M. I.; Marchand, N.; Marin, C.; Marmureanu, L.; Mihalopoulos, N.; Močnik, G.; Nęcki, J.; O'Dowd, C.; Ovadnevaite, J.; Peter, T.; Petit, J.-E.; Pikridas, M.; Matthew Platt, S.; Pokorná, P.; Poulain, L.; Priestman, M.; Riffault, V.; Rinaldi, M.; Różański, K.; Schwarz, J.; Sciare, J.; Simon, L.; Skiba, A.; Slowik, J. G.; Sosedova, Y.; Stavroulas, I.; Styszko, K.; Teinemaa, E.; Timonen, H.; Tremper, A.; Vasilescu, J.; Via, M.; Vodička, P.; Wiedensohler, A.; Zografou, O.; Cruz Minguillón, M.; Prévôt, A. S. H. European aerosol phenomenology − 8: Harmonised source apportionment of organic aerosol using 22 Year-long ACSM/AMS datasets. *Environ. Int.* **2022**, *166*, 107325, https://doi.org/10.1016/j.envint.2022.107325.

(8) Paatero, P.; Tapper, U. Positive matrix factorization: A non-negative factor model with optimal utilization of error estimates of data values. *Environmetrics* **1994**, *5*, (2), 111–126.

(9) Paatero, P. The multilinear engine—A table-driven, least squares program for solving multilinear problems, including the n-way parallel factor analysis model. *J. Comput. Graph. Stat.* **1999**, *8*, 854–888.

(10) Mohr, C.; Huffman, J. A.; Cubison, M. J.; Aiken, A. C.; Docherty, K. S.; Kimmel, J. R.; Ulbrich, I. M.; Hannigan, M.; Jimenez, J. L. Characterization of primary organic aerosol emissions from meat cooking, trash burning, and motor vehicles with high-resolution aerosol mass spectrometry and comparison with ambient and chamber observations. *Environ. Sci. Technol.* **2009**, *43*, 2443–2449.

(11) Canagaratna, M. R.; Onasch, T. B.; Wood, E. C.; Herndon, S. C.; Jayne, J. T.; Cross, E. S.; Miake-Lye, R. C.; Kolb, C. E.; Worsnop, D. R. Evolution of vehicle exhaust particles in the atmosphere. *J. Air Waste Manage. Assoc.* **2010**, *60*, 1192–1203.

(12) Cubison, M. J.; Ortega, A. M.; Hayes, P. L.; Farmer, D. K.; Day, D.; Lechner, M. J.; Brune, W. H.; Apel, E.; Diskin, G. S.; Fisher, J. A.; Fuelberg, H. E.; Hecobian, A.; Knapp, D. J.; Mikoviny, T.; Riemer, D.; Sachse, G. W.; Sessions, W.; Weber, R. J.; Weinheimer, A. J.; Wisthaler, A.; Jimenez, J. L. Effects of aging on organic aerosol from open biomass burning smoke in aircraft and laboratory studies. *Atmos. Chem. Phys.* **2011**, *11*, 12049–12064.

(13) DeCarlo, P. F.; Dunlea, E. J.; Kimmel, J. R.; Aiken, A. C.; Sueper, D.; Crounse, J.; Wennberg, P. O.; Emmons, L.; Shinozuka, Y.; Clarke, A.; Zhou, J.; Tomlinson, J.; Collins, D. R.; Knapp, D.; Weinheimer, A. J.; Montzka, D. D.; Campos, T.; Jimenez, J. L. Fast airborne aerosol size and chemistry measurements above Mexico City and Central Mexico during the MILAGRO campaign. *Atmos. Chem. Phys.* **2008**, *8*, 4027–4048.

(14) Alfarra, M. R.; Prevot, A. S. H.; Szidat, S.; Sandradewi, J.; Weimer, S.; Lanz, V. A.; Schreiber, D.; Mohr, M.; Baltensperger, U. Identification of the mass spectral signature of organic aerosols from wood burning emissions. *Environ. Sci. Technol.* **2007**, *41*, 5770–5777.

(15) Lin, C.; Ceburnis, D.; Hellebust, S. M.; Buckley, P.; Wenger, J. C.; Canonaco, F.; Prevot, A. S. H.; Huang, R.-J.; O'Dowd, C.; Ovadnevaite, J. Characterization of primary organic aerosol from domestic wood, peat, and coal burning in Ireland. *Environ. Sci. Technol.* **2017**, *51*, 10624–10632.

(16) Dall'Osto, M.; Ovadnevaite, J.; Ceburnis, D.; Martin, D.; Healy, R. M.; O'Connor, I. P.; Kourtchev, I.; Sodeau, J. R.; Wenger, J. C.; O'Dowd, C. Characterization of urban aerosol in Cork city (Ireland) using aerosol mass spectrometry. *Atmos. Chem. Phys.* **2013**, *13*, (9), 4997–5015.

(17) Mohr, C.; DeCarlo, P. F.; Heringa, M. F.; Chirico, R.; Slowik, J. G.; Richter, R.; Reche, C.; Alastuey, A.; Querol, X.; Seco, R.; Peñuelas, J.; Jiménez, J. L.; Crippa, M.; Zimmermann, R.; Baltensperger, U.; Prévôt, A. S. H. Identification and quantification of organic aerosol from cooking and other sources in Barcelona using aerosol mass spectrometer data. *Atmos. Chem. Phys.* **2012**, *12*, 1649–1665.

(18) Crippa, M.; DeCarlo, P. F.; Slowik, J. G.; Mohr, C.; Heringa, M. F.; Chirico, R.; Poulain, L.; Freutel, F.; Sciare, J.; Cozic, J.; Di Marco, C. F.; Elsasser, M.; Nicolas, J. B.; Marchand, N.; Abidi, E.; Wiedensohler, A.; Drewnick, F.; Schneider, J.; Borrmann, S.; Nemitz, E.; Zimmermann, R.; Jaffrezo, J. L.; Prévôt, A. S. H.; Baltensperger, U. Wintertime aerosol chemical composition and source apportionment of the organic fraction in the metropolitan area of Paris. *Atmos. Chem. Phys.* **2013**, *13*, 961–981.

(19) Ng, N.; Canagaratna, M.; Zhang, Q.; Jimenez, J.; Tian, J.; Ulbrich, I.; Kroll, J.; Docherty, K.; Chhabra, P.; Bahreini, R.; Murphy, S. M.; Seinfeld, J. H.; Hildebrandt, L.; Donahue, N. M.; DeCarlo, P. F.; Lanz, V. A.; Prévôt, A. S. H.; Dinar, E.; Rudich, Y.; Worsnop, D. R. Organic aerosol components observed in Northern Hemispheric datasets from Aerosol Mass Spectrometry. *Atmos. Chem. Phys.* **2010**, *10*, 4625–4641.

(20) Ramadan, Z.; Eickhout, B.; Song, X.-H.; Buydens, L. M. C.; Hopke, P. K. Comparison of positive matrix factorization and multilinear engine for the source apportionment of particulate pollutants. *Chemometr. Intell. Lab. Syst.* **2003**, *66*, 15–28.

(21) Liu, B.; Yang, J.; Yuan, J.; Wang, J.; Dai, Q.; Li, T.; Bi, X.; Feng, Y.; Xiao, Z.; Zhang, Y.; Xu, H. Source apportionment of atmospheric pollutants based on the online data by using PMF and ME2 models at a megacity, China. *Atmos. Res.* **2017**, *185*, 22–31.

(22) Huang, R. J.; Wang, Y.; Cao, J.; Lin, C.; Duan, J.; Chen, Q.; Li, Y.; Gu, Y.; Yan, J.; Xu, W.; Fröhlich, R.; Canonaco, F.; Bozzetti, C.; Ovadnevaite, J.; Ceburnis, D.; Canagaratna, M. R.; Jayne, J.; Worsnop, D. R.; El-Haddad, I.; Prévôt, A. S. H.; O'Dowd, C. D. Primary emissions versus secondary formation of fine particulate matter in the most polluted city (Shijiazhuang) in North China. *Atmos. Chem. Phys.* **2019**, *19*, 2283–2298.

(23) Ng, N. L.; Canagaratna, M. R.; Jimenez, J. L.; Chhabra, P. S.; Seinfeld, J. H.; Worsnop, D. R. Changes in organic aerosol composition with aging inferred from aerosol mass spectra. *Atmos. Chem. Phys.* **2011**, *11*, 6465–6474.

(24) Elser, M.; Huang, R.-J.; Wolf, R.; Slowik, J. G.; Wang, Q.; Canonaco, F.; Li, G.; Bozzetti, C.; Daellenbach, K. R.; Huang, Y.; Zhang, R.; Li, Z.; Cao, J.; Baltensperger, U.; El-Haddad, I.; Prévôt, A. S. H. New insights into $PM_{2.5}$ chemical composition and sources in two major cities in China during extreme haze events using aerosol mass spectrometry. *Atmos. Chem. Phys.* **2016**, *16*, 3207–3225.

(25) Duan, J.; Huang, R. J.; Lin, C.; Dai, W.; Wang, M.; Gu, Y.; Wang, Y.; Zhong, H.; Zheng, Y.; Ni, H.; Dusek, U.; Chen, Y.; Li, Y.; Chen, Q.; Worsnop, D. R.; O'Dowd, C. D.; Cao, J. Distinctions in source regions and formation mechanisms of secondary aerosol in Beijing from summer to winter. *Atmos. Chem. Phys.* **2019**, *19*, 10319–10334.

(26) Canonaco, F.; Crippa, M.; Slowik, J. G.; Baltensperger, U.; Prévôt, A. S. H. SoFi, an IGOR-based interface for the efficient use of the generalized multilinear engine (ME-2) for the source apportionment: ME-2 application to aerosol mass spectrometer data. *Atmos. Meas. Tech.* **2013**, *6*, (12), 3649–3661.

(27) Chen, B.; Andersson, A.; Lee, M.; Kirillova, E. N.; Xiao, Q.; Kruså, M.; Shi, M.; Hu, K.;

Lu, Z.; Streets, D. G.; Du, K.; Gustafsson, Ö. Source forensics of black carbon aerosols from China. *Environ. Sci. Technol.* **2013**, *47*, 9102–9108.

(28) Zhang, Y. L.; El-Haddad, I.; Huang, R. J.; Ho, K. F.; Cao, J. J.; Han, Y.; Zotter, P.; Bozzetti, C.; Daellenbach, K. R.; Slowik, J. G.; Salazar, G.; Prévôt, A. S. H.; Szidat, S. Large contribution of fossil fuel derived secondary organic carbon to water soluble organic aerosols in winter haze in China. *Atmos. Chem. Phys.* **2018**, *18*, 4005–4017.

(29) Zhang, Y. L.; Huang, R. J.; El Haddad, I.; Ho, K. F.; Cao, J. J.; Han, Y.; Zotter, P.; Bozzetti, C.; Daellenbach, K. R.; Canonaco, F.; Slowik, J. G.; Salazar, G.; Schwikowski, M.; Schnelle-Kreis, J.; Abbaszade, G.; Zimmermann, R.; Baltensperger, U.; Prévôt, A. S. H.; Szidat, S. Fossil vs. non-fossil sources of fine carbonaceous aerosols in four Chinese cities during the extreme winter haze episode of 2013. *Atmos. Chem. Phys.* **2015**, *15*, 1299–1312.

(30) Fang, W.; Andersson, A.; Zheng, M.; Lee, M.; Holmstrand, H.; Kim, S.-W.; Du, K.; Gustafsson, Ö. Divergent evolution of carbonaceous aerosols during dispersal of East Asian haze. *Sci. Rep.* **2017**, *7*, 10422, https://doi.org/10.1038/s41598-017-10766-4.

(31) Yan, C.; Zheng, M.; Bosch, C.; Andersson, A.; Desyaterik, Y.; Sullivan, A. P.; Collett, J. L.; Zhao, B.; Wang, S.; He, K.; Gustafsson, Ö. Important fossil source contribution to brown carbon in Beijing during winter. *Sci. Rep.* **2017**, *7*, 43182, https://doi.org/10.1038/srep43182.

(32) Liu, D.; Vonwiller, M.; Li, J.; Liu, J.; Szidat, S.; Zhang, Y.; Tian, C.; Chen, Y.; Cheng, Z.; Zhong, G.; Fu, P.; Zhang, G. Fossil and non-fossil fuel sources of organic and elemental carbon aerosols in Beijing, Shanghai and Guangzhou: Seasonal carbon-source variation. *Aerosol Air Qual. Res.* **2020**, *20*, 2495–2506.

(33) Ni, H.; Huang, R. J.; Cosijn, M. M.; Yang, L.; Guo, J.; Cao, J.; Dusek, U. Measurement report: Dual-carbon isotopic characterization of carbonaceous aerosol reveals different primary and secondary sources in Beijing and Xi'an during severe haze events. *Atmos. Chem. Phys.* **2020**, *20*, 16041–16053.

(34) Kirillova, E. N.; Andersson, A.; Han, J.; Lee, M.; Gustafsson, Ö. Sources and light absorption of water-soluble organic carbon aerosols in the outflow from northern China. *Atmos. Chem. Phys.* **2014**, *14*, 1413–1422.

(35) Vu, T. V.; Shi, Z.; Cheng, J.; Zhang, Q.; He, K.; Wang, S.; Harrison, R. M. Assessing the impact of clean air action on air quality trends in Beijing using a machine learning technique. *Atmos. Chem. Phys.* **2019**, *19*, 11303–11314.

(36) Grange, S. K.; Carslaw, D. C.; Lewis, A. C.; Boleti, E.; Hueglin, C. Random forest meteorological normalisation models for Swiss PM_{10} trend analysis. *Atmos. Chem. Phys.* **2018**, *18*, 6223–6239.

(37) Wang, Y.; Huang, R.-J.; Xu, W.; Zhong, H.; Duan, J.; Lin, C.; Gu, Y.; Wang, T.; Li, Y.; Ovadnevaite, J.; Ceburnis, D.; O'Dowd, C. Staggered-peak production is a mixed blessing in the control of particulate matter pollution. *npj Clim. Atmos. Sci.* **2022**, *5*, 99, https://doi.org/10.1038/s41612-022-00322-x.

(38) Wang, S.; Su, H.; Chen, C.; Tao, W.; Streets, D. G.; Lu, Z.; Zheng, B.; Carmichael, G. R.; Lelieveld, J.; Pöschl, U.; Cheng, Y. Natural gas shortages during the "coal-to-gas" transition in China have caused a large redistribution of air pollution in winter 2017. *Proc. Natl. Acad. Sci.* **2020**, 117, 31018–31025.

(39) Zhao, S.; Hu, B.; Gao, W.; Li, L.; Huang, W.; Wang, L.; Yang, Y.; Liu, J.; Li, J.; Ji, D.; Zhang, R.; Zhang, Y.; Wang, Y. Effect of the "coal to gas" project on atmospheric NO_X during the heating period at a suburban site between Beijing and Tianjin. *Atmos. Res.* **2020**, 241, 104977.

(40) Ni, H.; Huang, R. J.; Cao, J.; Guo, J.; Deng, H.; Dusek, U. Sources and formation of carbonaceous aerosols in Xi'an, China: Primary emissions and secondary formation constrained by radiocarbon. *Atmos. Chem. Phys.* **2019,** *19*, 15609–15628.
